# Current hydrogel advances in physicochemical and biological response-driven biomedical application diversity

**DOI:** 10.1038/s41392-021-00830-x

**Published:** 2021-12-16

**Authors:** Huan Cao, Lixia Duan, Yan Zhang, Jun Cao, Kun Zhang

**Affiliations:** 1 Department of Nuclear Medicine, West China Hospital, and National Engineering Research Center for Biomaterials, Sichuan University, 610064 Chengdu, P. R. China; 2 Department of Medical Ultrasound and Central Laboratory, Shanghai Tenth People’s Hospital, Tongji University School of Medicine, No. 301 Yan-chang-zhong Road, 200072 Shanghai, People’s Republic of China; 3 School of Materials Science and Engineering, Nanyang Technological University, 50 Nanyang Avenue, Singapore, 639798 Singapore

**Keywords:** Translational research, Biomaterials, Biophysics

## Abstract

Hydrogel is a type of versatile platform with various biomedical applications after rational structure and functional design that leverages on material engineering to modulate its physicochemical properties (e.g., stiffness, pore size, viscoelasticity, microarchitecture, degradability, ligand presentation, stimulus-responsive properties, etc.) and influence cell signaling cascades and fate. In the past few decades, a plethora of pioneering studies have been implemented to explore the cell–hydrogel matrix interactions and figure out the underlying mechanisms, paving the way to the lab-to-clinic translation of hydrogel-based therapies. In this review, we first introduced the physicochemical properties of hydrogels and their fabrication approaches concisely. Subsequently, the comprehensive description and deep discussion were elucidated, wherein the influences of different hydrogels properties on cell behaviors and cellular signaling events were highlighted. These behaviors or events included integrin clustering, focal adhesion (FA) complex accumulation and activation, cytoskeleton rearrangement, protein cyto-nuclei shuttling and activation (e.g., Yes-associated protein (YAP), catenin, etc.), cellular compartment reorganization, gene expression, and further cell biology modulation (e.g., spreading, migration, proliferation, lineage commitment, etc.). Based on them, current in vitro and in vivo hydrogel applications that mainly covered diseases models, various cell delivery protocols for tissue regeneration and disease therapy, smart drug carrier, bioimaging, biosensor, and conductive wearable/implantable biodevices, etc. were further summarized and discussed. More significantly, the clinical translation potential and trials of hydrogels were presented, accompanied with which the remaining challenges and future perspectives in this field were emphasized. Collectively, the comprehensive and deep insights in this review will shed light on the design principles of new biomedical hydrogels to understand and modulate cellular processes, which are available for providing significant indications for future hydrogel design and serving for a broad range of biomedical applications.

## Introduction

Hydrogels are a class of water-swollen three-dimensional (3D) polymer network featuring tunable physicochemical properties that are demanded to satisfy the specific requirements under different conditions. As a type of promising materials, they have been extensively applied in the biomedical field ranging from physiological and pathological mechanism studies, to tissue regeneration and disease therapies.^1–4^ Generally, the properties of as-prepared hydrogel scaffolds are determined by the material composition/concentration, cross-linking methods/density and fabrication approaches. Typically, the variable compositions including collagen, gelatin, and polyethylene glycol (PEG)-based hydrogels correspond to fibrous, macroporous, and nanoporous architectures, respectively.^5^ Notably, physical or chemical cross-linking protocols could also alter mechanical properties of hydrogels, e.g., physical cross-linking represented by hydrophobic interactions, hydrogen bonding, polymerization entanglement, π–π stacking, etc., usually suffers from poor mechanical strength, while covalent cross-linking (e.g., free radical polymerization, enzyme-induced cross-linking, etc.) will bring about high mechanical properties.^6–8^ As well, high cross-linking density also favors dense structure and enhanced stiffness. It is worth noting that the fabrication approaches (in situ gelation,^9^ electrospinning,^10^ micropatterning,^11^ 3D bioprinting,^12^ microfluidics,^13^ etc.), also matter for hydrogel properties and determine their applications. As a paradigm, cell attachment sites in hydrogels could be finely tuned via micropatterning and microfluidic strategies, which is preferable for exclusively studying any cue’s effects on cell biology. Hydrogels featuring in situ gelation are appropriate for subcutaneous injection.

The interaction between cell and hydrogel is complex and dynamic, which exerts significant impacts on tissue physiological (e.g., cell spreading,^14^ proliferation,^15^ migration,^16^ stemness,^17^ differentiation,^18^ etc.) and pathological processes, such as cell apoptosis,^19^ fibrosis,^20^ immunological rejection,^21^ etc. Regarding this, a comprehensive and deep understanding of cell–hydrogel interaction especially at the molecular level is of great importance, which is helpful for guiding the rational design of hydrogels and facilitating their clinic translation in the future. In general, once exposed to an external hydrogel matrix, cells will respond to the static physicochemical cues of hydrogels (stiffness,^22,23^ pore size,^24,25^ viscoelasticity,^26–28^ microarchitecture,^29,30^ degradability,^17,31^ chemical surface,^32–34^ etc.) and then switch these cues into biochemical signals to tune their biology and homeostasis. Accumulative evidences have shown that the harbored cells can in real time perceive and respond to the surrounding microenvironment changes induced by external stimuli in a spatial- and temporal-controlled manner.^35–39^ By adjusting intracellular signaling events such as dynamical integrin clustering regulation, focal adhesion (FA) complex accumulation and activation, cytoskeleton rearrangement, environmental cues-responsive proteins (e.g., Yes-associated protein (YAP), transcriptional co-activator with a PDZ-binding motif (TAZ), catenin, etc.) activation, gene expression, etc., cells displayed different biological characteristics and behaviors.^40,41^ Intriguingly, the hydrogels can be gradually degraded over time or/and metabolized by cells, further affecting cell behaviors.^42^

Currently, hydrogels have been explored and used in various biomedical applications according to their variable physicochemical, biological, and structural characteristics. One of the most well-known fields is esthetic medicine, and different commercial hydrogel products have been employed as fillers, e.g., hyaluronic acid-based hydrogel.^43^ Moreover, hydrogels have been extensively used as 3D models of different diseases (e.g., tumor model,^44^ tissue fibrosis models,^20^ corneal disease model,^45^ nerve disease model,^46^ inflammatory bowel disease,^47^ etc.) for pathogenesis study or high-throughput drug screening. Due to the in vivo tissue stroma matrix-mimicked property, hydrogels are favorable for cell encapsulation and expansion in vitro and in vivo, enabling high-efficient tissue regeneration and cancer therapy. For instance, various cells (stem cells,^48,49^ islet cells,^50^ hepatocytes^51^, endothelial cells (ECs),^52^ etc.) that were encapsulated in hydrogels could propagate and concurrently maintain functional characteristics in vitro. Afterwards, they were transferred into the designed disease site and act as protein/factor factory to sustainably promote and induce tissue regeneration and repair. Especially when carrying immune cells (e.g., T cells,^53^ natural killer (NK) cells,^54^ dendritic cells (DCs),^55^ macrophages,^56^ etc.), hydrogels can serve as immune niches for cancer immunotherapy. In particular, hydrogels could be engineered into cancer vaccines via loading with antigen, adjuvant (e.g., granulocyte macrophages colony-stimulating factor), or chemoattractant, boosting the systematic antitumor immunity.^57,58^ Similar to other nanocarriers for drug delivery, hydrogels are also regarded as ideal drug carriers for controlled and sustainable release at sites of interest and treatment efficiency evaluation.^59–62^ Moreover, when uniting with functional units, hydrogels are allowed to associate with bioimaging,^63^ biosensor,^64,65^ and conductive wearable/implantable biodevices.^66,67^

In terms of clinical translation, many facial corrections and esthetic hydrogel-based products have been approved by Food and Drug Administration (FDA).^68,69^ Some clinical trials also have confirmed the effectiveness of hydrogel-based therapy in various areas: knee osteoarthritis, spinal fusion, and spine, oral–maxillofacial and orthopedic trauma surgeries, advanced heart failure, type 2 diabetes, chronic kidney disease, etc.^70^ However, there are still many issues and challenges that needed to be addressed for more extensive and efficient biomedical applications, and more considerations are necessary for clinical translation.

**Fig. 1:**
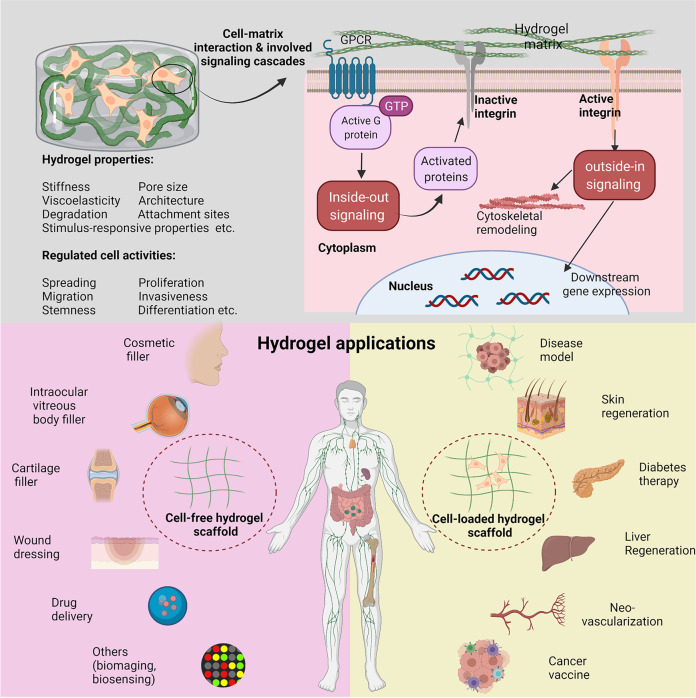
Schematic images for indicating the interactions between cell and hydrogel matrix, uncovering the influences of hydrogel physicochemical properties on cell biology via correspondingly triggering signaling cascades (e.g., inside-out and outside-in signaling), and illustrating various hydrogel biomedical applications of cell-free and cell-loaded hydrogels

Collectively, in this review, we summarized and discussed the current advances in the development of hydrogels for biomedical applications and their correlations with biological responses and related signaling cascades (Fig. 1). First, we gave a glimpse at the category of hydrogels and outlined hydrogel construction including hydrogel materials and techniques, wherein distinct physicochemical properties were underlined, as shown in Table 1. Subsequently, we surveyed the influences of hydrogel properties (e.g., stiffness, viscoelasticity, microarchitecture, pore size, degradability, cell attachment sites, stimulus-responsive properties, etc.) on cell biological responses. More significantly, we provided critical insights into cell behaviors and signaling transduction (e.g., integrin clustering, FA complex accumulation and activation, cytoskeleton rearrangement, environmental-responsive protein cyto-nuclei shuttling and activation, gene expression, etc.). Based on these comprehensive reviewing, the potential applications of hydrogels in vitro and in vivo, including high-throughput drug screening, tissue engineering, diseases therapy, gene therapy, drug delivery, etc., were presented with an emphasis on how the physicochemical or biological responses determined the application selection and design requirements of hydrogels. In the end, the clinical translation potential of hydrogels as well as the decisive factors were analyzed, and the unresolved challenges and future perspectives in this area were simultaneously discussed.

**Table 1:** Compositions and physicochemical properties of prepared hydrogels for determining biomedical application diversity

	Hydrogel material	Cross-linking method	Bioactivity	Degradability	Network pore size	Viscoelasticity	Stiffness	References
Natural material	Collagen	•pH and temperature •TG, Ca^2+^ •PEG-diNHS •Methacrylation/photopolymerization	Col-derived triple-helical ligands (e.g., GxOGER)—β1-containing integrins, RGD peptides	Collagenase, MMPs, plasmin, pepsin	Fibrous	Viscoelastic	•10–250 Pa (*G*’)	^74,75^
	Gelatin	•TG, Ca^2+^ •Methacrylation/photopolymerization •ferulic acid (FA) conjugation/Laccase (O_2_)	RGD—α5β1 and αvβ3	Collagenase, MMPs, plasmin, pepsin	Macroporous	Viscoelastic	•11–1800 Pa (*G*’)	^79^
	Hyaluronic acid (HA)	•Acrylation/photopolymerization •Host–guest interaction	Specially reacts with CD44 receptor	Hyaluronidase, MMPs	–	Viscoelastic	•1.3–10.6 kPa (*E*)	^81–83^
	Alginate	•Divalent cations (e.g., Ca^2+^ or Ba^2+^) •Oxidation + gelatin	/	Chelator (e.g., sodium citrate, EDTA, etc.)	Nanoporous	Viscoelastic	•0.5–3 kPa (*G*’)	^5,84^
	Fibrin	•Thrombin/Ca^2+^ •PEG-diNHS	RGD—α5β1 and αvβ3	chymotrypsin, actinase, carboxylase, etc.	Fibrous	Viscoelastic	•0–8 kPa (*G*’)	^85^
	Chitosan	•Glutaraldehyde •Genipin •Diisocyanate	/	Hydrolysis	Macroporous	Viscoelastic	•2–30 kPa (*G*’)	^86^
	Agarose	•Temperature	/	Hydrolysis	–	Viscoelastic	•1–120 kPa (G*)	^87^
	Polypeptide	•Self-assembly	-	Enzyme	Fibrous	Viscoelastic	•500–2500 Pa	^89–91^
	DNA	•Base pairing	/	Nuclease	–	Viscoelastic	•4–23 kPa (*E*’)	^92,93^
Synthetic material	PEG	•Acrylation/photopolymerization •Thiolation	/	Hydrolysis	–	Elastic	•1–1200 kPa (compression stress)	^95^
	PVA	•Calcium gluconate •Glutaraldehyde, etc.	/	Hydrolysis	–	Elastic	•3.7–30.2 kPa	^96–98^
	PCL	•Temperature	/	Hydrolysis	–	Elastic	•~5 MPa (compressive strength)	^101^
	Polyacrylamide	•Free radical copolymerization	/	Hydrolysis	–	Elastic	•0.1–740 kPa	^99,100^
	Polyurethane (PU)	•Temperature	/	Hydrolysis	–	Elastic	•0.68–2.4 kPa (*E*’)	^102,103^

Note: / indicates none; - represents depending

## Hydrogel materials and scaffold fabrication strategies

**Fig. 2:**
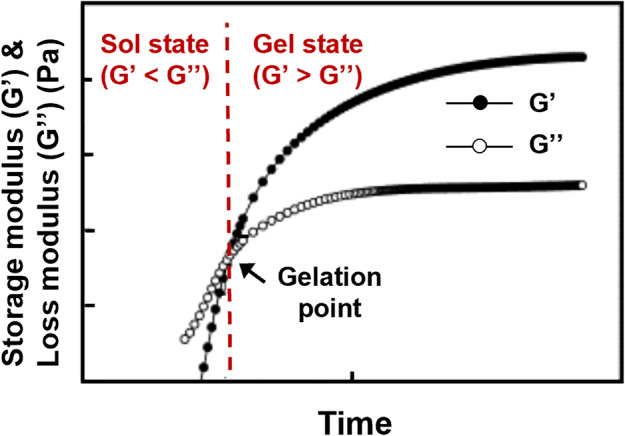
Rheological characterization of solution to gelation (sol–gel) transition process of hydrogel precursor. Modified from ref. ^71^. Copyright 2006, Elsevier

Hydrogel is a 3D polymeric network with high water content (>90%), whose rheological properties (i.e., the law of flow or deformation of materials under external factors (e.g., stress, strain, temperature, etc.) in relation to time) can be well characterized via oscillation mode on a rheometer. In this measurement, the obtained storage modulus (*G*’) and loss modulus (*G*”) reflect the elastic and viscous moduli of hydrogels, respectively. In particular, the cross point of *G*’ and *G*” was determined as the phase transition point (gelation point) of hydrogels (Fig. 2),^71^ which can be identified as one typical hallmarker for distinguishing hydrogels from other liquid materials (e.g., polymer melts or solutions, suspensions, etc.) or films.

### Hydrogel classification and physicochemical property

Hydrogels can be classified into two types, i.e., natural hydrogels and synthetic hydrogels. Natural hydrogels, such as collagen, gelatin, hyaluronic acid (HA), chitosan, etc., always have good biocompatibility and biodegradability. However, their mechanical properties such as rigidity and stretchability are poor, which restrict their application. In contrast, the synthetic hydrogels (e.g., PEG derivatives, polycaprolactone (PCL), polyvinyl alcohol (PVA), etc.) are chemically cross-linked with relatively higher mechanical properties, which can withstand strong mechanical loads despite the fact that they suffer from poor biological activity and unsatisfactory biocompatibility. In recent years, some novel self-designed polypeptide and DNA hydrogels are also extensively investigated. Overall, a summary that exemplifies the typical hydrogel materials and their physicochemical properties is provided (Table 1) so as to offer a systematic impression on hydrogels.

#### Natural materials

Collagen is the most abundant component of extracellular matrix (ECM), and the extracted collagen could be spontaneously transformed into hydrogels via pH and temperature-dependent self-fibrogenesis processes under physiological condition.^72^ Collagen fibrils and fibers with only hundreds of nano-meter thickness are enriched in cell adherence peptide ligands such as collagen triple-helical ligands (GxOGER) and arginine–glycine–aspartic acid (RGD), and these peptides can bind with cell membrane receptors, such as α1β1 and α2β1 integrins.^73,74^ As a result, collagen hydrogels can be recognized as a biomimetic substance to physiologically mimic the cellular microenvironment in vivo. However, the weak mechanical strength during high cell traction and batch-to-batch difference considerably discourages its application. Therefore, myriad efforts have been made to increase its mechanical property and stability via chemical cross-linking methods, typically represented by glycation and enzyme-mediated cross-linking.^75^ In addition, some studies also reported that the synthesized PEG conjugated to di(succinic acid *N*-hydroxysuccinimide ester) could work as a cross-linker for enabling the collagen fiber interconnection via amine cross-linking, consequently resulting in varied elastic moduli (*E*_0_) ranging from 0.7 to 4.0 kPa.^76^ Intriguingly, methylated collagen hydrogels were also developed, and the gelation could be initiated by photo irradiation,^77^ which paves the way to the new application of collagen hydrogels in the future.

Gelatin is the product deriving from the moderate hydrolysis and thermal denaturation of collagen, but it fails to be equipped with the triple helix structure. Inspired by it, it is also a desired substitute for collagen due to the identical molecule composition with collagen.^78^ Gelatin is also biodegradable by cell-derived enzymes (e.g., matrix metalloproteinases (MMPs)), and this behavior will benefit the cell-mediated stroma remodeling via ECM protein deposition and cell contraction force-induced network rearrangement. In an attempt to develop chemical cross-linking approaches to reinforce the mechanical properties, enzyme-induced (e.g., transglutaminase) cross-linking and photo-induced covalent cross-linking that correspond to pure and methacrylate-modified gelatin, respectively, are dominant.^79^ Notably, the network architecture of gelatin and collagen hydrogel scaffolds are dramatically different, corresponding to macroporous and fibrous structures, respectively, which could modulate different cell behaviors and fates via mechanotransduction signaling and benefit different applications.^5^ However, the composition used is not the only cause of the final scaffold architecture. Actually, the fabrication process and modification approaches also take the responsibility for the eventual scaffold architecture.

HA is a highly hydrophilic ECM component with cell-binding sites for various cell types that overexpress receptor CD44, which, thus, can exert robust influences on cell migration, differentiation, etc. For instance, it is reported that HA-based hydrogels have been documented to trigger the spontaneous polarity of M2-like monocyte/macrophage via CD44-mediated signal transducer and activator of transcription 3 (STAT3) activation in THP-1 cells.^34^ Additionally, the molecular weight (MW) of HA is a determinant factor for different cell behaviors due to the inconsistent CD44 clustering.^80^ HA with higher Mw (∼10^7^ Da) (nHA) than smaller segments (oHA) is found to inhibit angiogenesis and inflammatory, and vice versa. In comparison, oHA is adept at stimulating the proliferation of ECs through regulating G1 phase of cell cycle progression in a distinct way. In general, HA is disabled to be autonomously cross-linked, and different chemical modifications of HA polymer chains including hydrazide-functionalized HA,^81^ thiolated HA,^82^ methacrylated HA,^83^ etc., are recommended before designing appropriate hydrogels aiming at different specific applications.

Alginate is usually believed to be a bioinert material composing of homopolymeric blocks of (1-4)-linked β-D-mannuronate (M) and C-5 epimer α-L-guluronate (G) residues. The G residues can be cross-linked by divalent cations like Ca^2+^ to form an egg-box structure, giving birth to alginate hydrogels.^84^ More interestingly, the chelated Ca^2+^ within hydrogels could be easily deprived of by some highly coordinated chelators such as sodium citrate or ethylene diamine tetraacetic acid, resulting in hydrogel decomposition.^84^ The cross-linked alginate hydrogel displayed a nanoporous structure similar to the basement membrane, which could impose physical confinement on harbored cells and influence cell spreading, migration, differentiation, etc.^5^ Moreover, alginate hydrogels constructed with different MWs showed tunable viscoelasticity and stress relaxation time, which further benefited cell-contraction force-induced ligands (e.g., RGD) clustering and affected FA complexes’ formation and mechanosensitive proteins’ activation (e.g., YAP).^14,27^

Besides above common and extensively used hydrogels, there are many other natural hydrogels, e.g., silk fibroin,^85^ chitosan,^86^ agarose,^87^ protein (e.g., bovine serum albumin)^88^ and polypeptide^89–91^ and DNA^92,93^ hydrogels, etc., as summarized in Table 1, which also show high potentials for various biomedical applications. More significantly, these hydrogels attract increasing interests, among which polypeptide hydrogels,^90,91^ DNA hydrogels,^92,93^ etc., merit more attentions due to their extraordinary genetic information. Polypeptides are the intermediate products of proteolysis, and their amphiphilic chains can be self-assembled into a 3D hydrogel network via hydrogen bonds and electrostatic interactions. Thus, they share high biocompatibility, and can be endowed with stimulus-responsive property and tunable mechanical property, holding great potentials in tissue engineering, drug delivery, and biosensing.^91^ Similarly, DNA hydrogels can also be self-assembled, but this process obeys Watson–Crick base-paring rules. It allows the accurate modulation of molecular structure and function tailoring in DNA hydrogels, harvesting unexpected excellences such as indispensable genetic function, extensive bio-compatibility, precise molecular recognition, multifunctionality, and convenient programmability.^94^

#### Synthetic materials

PEG derivatives are the most extensively used synthetic biocompatible hydrogel materials because of their ease of preparation, relatively low cost, non-biodegradability, the ease of chemical modification and tunable mechanical properties.^1^ The most common approach for yielding PEG hydrogel is photopolymerization of poly(ethylene glycol) diacrylate (PEGDA) chains. However, pristine PEG derivatives are bioinert and thus are unable to support cell adhesion. Therefore, to render the PEG hydrogel bioactive and biodegradable, chemical modification is imperative, where cell integrin-binding motifs and MMPs are typically conjugated onto the backbone of the PEG polymer chains.^95^

PVA is another synthetic hydrogel with high biocompatibility for medical treatment, e.g., soft contact lenses, eye drops, tissue adhesion barrier, artificial joints, artificial kidney membrane, etc.^96,97^ However, the inherently low mechanical property severely discourages the development of PVA hydrogels. As a result, different approaches have been established to heighten their mechanical properties, such as double cross-linking method and multi-walled carbon nanotube doping.^98^

As the classic temperature-sensitive hydrogels, poly(*N*-isopropylacrylamide) (PNIPAAm) and polyacrylamide are two water-soluble polymers that are produced through free radical polymerization of acrylamide (AAm) monomer alone or combination with acrylic acid (AAc). AAM/AAc ratio can be tuned to manipulate the lower critical switch temperature of hydrogels from liquid to solid state. Currently, the investigations on the two hydrogels have been fully carried out, and they were found to play an important role in drug delivery, mesenchymal stem cell (MSC) carrier, and other tissue engineering.^99,100^ However, the poor biodegradability and potential toxic effect of such two hydrogels on cells still impeded their development and application in biomedical field.

As well, there are a series of other synthetic hydrogels for certain biomedical applications. Typically, PCL has been widely used as tissue engineering scaffold material because it is soft, easy to manufacture, and has a long biodegradable period and an excellent biocompatibility.^101^ Polyurethane (PU) is a class of synthetic material obtained by the reaction of polyol and isocyanate. The mechanical properties of PU hydrogels can be easily and finely tuned by altering the chemical structure of adopted polyols and isocyanate.^102,103^ Actually, to obtain more functions and elevate their performance, various composite hydrogels consisting of multiple hydrogels have also been developed and employed to regulate porosity, pore size, mechanical strength, etc.^104–106^ As a paradigm, three hydrogel components, i.e., PEG, PNIPAAm, and chitosan that are usually used individually, respectively, can be integrated to yield the physical cross-linked chitosan–PEG–PNIPAAm composite hydrogel. This composite hydrogels were endowed with the pH and temperature sensitivities as well as enhanced and tunable mechanical properties via varying PEG MW compared to their personal component alone.^105^

### Preparation strategies and related characteristics of hydrogel scaffolds

The preparation method of hydrogel scaffolds is another key point that can decide their application field. Several approaches, such as in situ gelation, electrospinning, micropatterning, bioprinting, microfluidics, etc., have been developed over the years to generate hydrogel scaffolds with distinct properties and features to satisfy the requirements of their specific biomedical application.

#### Preparation methods and properties of hydrogel scaffolds

##### In situ gelation

In situ injectable hydrogels have been intensively investigated because of their easy-to-tackle property and the ease of encapsulating bioactive ingredients and/or cells into hydrogel precursor solution. After simple physical mixing for loading exogenous substances and injection at the desired lesion, the spontaneous or external stimuli-initiated cross-linking process representing gelation was carried out.^107,108^ There are several advantages for in situ gelated hydrogels. First, hydrogels can be delivered to the desired sites through minimally invasive injection and allow enhanced cell or/and drug retention at the site of injection, which is expected to improve patient compliance and curative efficacy. Second, in situ gelation can protect sensitive drugs such as proteins, genes, growth factors, etc., from enzyme biodegradation.^108^

Generally, there are three strategies to synthesize in situ hydrogels. One strategy is the polymerization of small molecules via chemical cross-linking (e.g., enzyme-induced cross-linking, high energy radiation, Diels–Alder “click chemistry”, photo-activated cross-linking, etc.) in the presence of initiators and cross-linkers.^109^ Of note, ultraviolet (UV) is the most potent trigger with the highest energy to evoke free radical generation, efficiently initiating hydrogel cross-linking. However, the limited tissue penetration depth of UV light denotes that current hydrogels (e.g., PEGDA) via UV-initiated cross-linking are applied only in in vitro tests. The second strategy is the direct cross-linking of either natural or synthetic hydrophilic polymers, which can be triggered by in vivo stimuli such as temperature, pH, enzyme, redox, and other factors. Notably, the gelation time in vivo should be taken into serious consideration for in situ injection application since too long gelation time would result in drug or cell leakage out of hydrogels to pervade across the whole disease site during cross-linking process, consequently reducing the therapeutic outcome.^110^ Up to now, different novel strategies have been developed to accelerate the gelation process in vivo.^111^ Intriguingly, self-healing as the third pathway that usually occur to self-healable hydrogels has emerged as a promising strategy for in situ gelation because of its dynamic and reversible cross-linking bonds, such as dynamic covalent bonds (e.g., Schiff base) and physical bonds (e.g., hydrogen bond).^112–114^ Generally, the drug or cell-accommodated self-healing hydrogels could be constructed in vitro, followed by injection into the targeted site. Due to the motion of polymer chains and the dynamic variation of cross-linking bonds, the injected hydrogel pieces could re-form the integral structure with unimpaired mechanical properties in situ, circumventing the injection problem of hydrogel precursor solution, namely low gelation rate.

##### Freeze drying

Freeze drying is an important approach for hydrogel scaffold construction. In general, the as-prepared hydrated or freeze-dried hydrogels after hydrogel precursor cross-linking can be directly used as scaffolds according to their different purposes. During freeze-dried hydrogel construction, the detailed parameters, such as temperature, freezing–melting times, etc., will significantly affect their physical properties (e.g., microarchitecture, swelling ratio, degradation, etc.) of the as-prepared scaffold via disturbing the evolution process of inner crystal.^115–117^ For instance, the decrease of freezing temperature from −10 to −70 °C could lead to the decreased average pore size of resulting scaffolds from 325 to 85 µm. The phenomenon is probably attributed to higher temperature that would lead to bigger ice crystal inside scaffold.^118^ As documented, pore size increase would further affect water uptake by scaffolds, consequently leading to elevations of swelling ratio and degradation rate as well as mechanical properties. Concurrently, the increased pore size would also accelerate the diffusion rate of encapsulated active molecules within scaffolds. For cell culturing, large pore size-induced flat surface could promote cell adhesion on these surfaces and simultaneously inhibit cell migration into scaffolds. The most important advantages of freeze-dried hydrogels are the relatively easy to store and long shelf-life. Despite this, the practical application field of as-prepared scaffolds is limited to invasive applications as implants or soft wound dressing. Inspiringly, different strategies have also been developed nowadays for preparing hydrogel scaffolds with more uniform pores,^119,120^ which are expected to expand their application.

##### Electrospinning

Electrospinning produces fibers with designed size and orientation by extracting viscoelastic polymers from a spinneret and subsequently depositing them on a collector plate. The fabrication mechanism and process have been clarified in other reviews. It has been documented that the process and the final fiber morphology associated with fiber diameter or orientation are susceptible to concentration, conductivity, viscosity, MW, solvent volatility, and molecular structure of the polymer solution.^121^ Regarding this, meticulous and adequate considerations are needed before fabricating hydrogel fiber via electrospinning technology.

Electrospinning scaffold has been widely used for biomedical applications, such as tissue engineering, drug delivery, etc.^122^ Researchers have reported a series of electrospun polyaniline–gelatin fiber scaffolds for cardiac tissue engineering, and these scaffolds could promote the adhesion and proliferation of cardiac myoblasts.^123^ In addition, the polymer nanofibers obtained via electrospinning could be weaved into surfaces, and these coated surfaces could serve as substrates to study cell responses to variable underlying topographies and chemistry surfaces. Typically, glioma cells cultured on PCL fibrous scaffolds showed elongated morphology and increased migration potential in white matter tissue, which could be attributed to JAK/STAT signaling activation. Meanwhile, cancer cell migration has been validated to depend on myosin II rather than stress fiber, and this behavior is opposite to those cells cultured on the two-dimensional (2D) substrate.^124^

Nanofibrous scaffolds were also used to deliver cell for enabling cell therapy against cancer. Bago et al. implanted a poly-lactic acid electrospinning scaffold containing MSCs into the tumor resection-left cavity. The scaffold implant could stimulate MSCs to release TRAIL antitumor protein for shrinking the volume of glioblastoma (GBM) xenograft and inhibiting its recurrence.^125^ Besides cell delivery, the electrospinning scaffolds that served as drug delivery systems have been widely explored. So far, many therapeutic drugs include small molecule drugs and biological substances, such as antibiotics, proteins, DNA, RNA, and growth factors, could be integrated into electrospinning fibers by encapsulation in the electrospinning process.^126^ The distinctive synthetic method and morphology determine that electrospinning nanofiber scaffolds are suitable for local administration, percutaneous administration, and oral administration, among which short nanofibers/fragments could be used for local injection to the lesion site with minimal invasion. In particular, the emergence of stimulus-responsive nanofibers in recent years furnished new approaches to the spatiotemporally controlled drug release.^127^

##### Micropatterning

Micropatterning technology is specially developed to control the surface geometry and chemistry state of cell-adhered area. Therefore, this method is extremely appropriate for statistically studying and analyzing the influences of environmental cues on cell biology behaviors, including cellular cytoskeleton dynamic rearrangement, polarity, cell mitosis, migration, differentiation, etc.^128,129^ When a pattern is formed at the subcellular-to-unicellular level, cell diffusion is impeded. Subsequently, the adhered cells are spontaneously driven to rearrange their cytoskeletons into designed spaces.^130^ At the multicellular scale, micropatterns are used to form microscale islands, making cells shaped into sheets with a specific shape, polarization and signal transduction.^131^ Monzo et al. created linear trajectories composed of laminin-coated micropatterns to mimic the vascular system for studying cell migration behavior in vitro. Results showed that GBM cells displayed saltatory migration manner similar to their in vivo motion, which was a result of combined actions of the microtubule-dependent polarization, contractile actin bundles and dynamic paxillin-containing adhesions. Interestingly, the formin inhibition led to muted GMB migration on micropatterns, indicating that formins like FHOD3 were involved in the enhanced migration.^132^

Moreover, with utilizing the micropatterning strategy, 3D microwell arrays can be obtained for generating multicellular spheroids. For instance, researchers have reported a case where micropatterning was used to yield a micro-coculture platform. In this platform, breast tumoroids were seeded inside microwells that were surrounded by the stromal cells (3T3-L1 adipocyte progenitor cells)-laden hydrogel matrix. The result showed that cancer cells could closely interact with the surrounding stroma cell and affect their differentiation into adipocytes in the presence of inducement media. More interestingly, stroma cell adipogenesis would be inhibited when the matrix stiffness increased from 200 Pa to 3 kPa. This result indicated that the stromal–cancer interactions were highly dependent on ECM stiffness, which provided significant indications for cancer therapeutic strategy establishment.^133^

Collectively, the hydrogel micropatterning technique is a powerful tool to engineer hydrogels into desired motifs or patterns for making cell study (e.g., signaling transduction, morphology alteration) easier. However, some concerns such as poor spatial resolution and pattern repeatability, high cost and complex fabrication process remain unresolved, which assuredly narrow their application domain and discourage their popularization.

##### Bioprinting

Bioprinting is established based on the assumption that a precise arrangement of cells can send physiological signals to produce functional tissues. It can allow hydrogel scaffolds to combine with objective cells, and then they are jointly manufactured into designed shapes through computer control.^134,135^ This method exhibits many advantages, e.g., process simplicity, low cost, and minimized waste. More inspiringly, it offers the ability to continuously and adaptably evolve, which makes this technology become a very powerful tool for revolutionizing our ability to iterate design in ways that are previously impossible.

Basically, there are two mainstream strategies for bioprinting: (1) one-step bioprinting manufacturing, wherein cells are encased in hydrogels and afterwards printed directly into structures; and (2) two-step bioprinting, wherein hydrogel materials are pre-printed into desired structures, followed by cell spreading onto the pre-printed scaffolds. Nowadays, various diseases/tissue models (e.g., neural tissue,^136^ tumor,^137^ etc.) or scaffold materials (e.g., bone substitutes^138^) based on 3D printing technology have been developed for tissue engineering and drug test. In a study conducted by Dai et al., 3D bio-printed brain tumor models constructed by glioma stem cells showed more robust resistance to chemotherapeutic agents compared to standard 2D cell models.^137^ This evident therapeutic difference between 2D and 3D platforms could explain the previous failures of many drug translations in clinics that mainly depended on lab data from 2D testing model, which thus highlighted the importance of biomimetic 3D models for drug test.

Nowadays, great efforts have been made to improve the resolution of 3D printing, and the reduced feature size can improve the fidelity of a bio-printed structure and simulate native tissues to regenerate. These advances will enable the perfect integration of hydrogels with 3D printing to make more contributions to multiple aspects of the biomedical field. However, rational design of biocompatible and printable bio-inks for 3D printing is still the major challenge, especially in the case of printing complex cell-loaded 3D structures for functional tissue construction. Thus, it needs more efforts to facilitate the extensive applications of bio-printing technology.

##### Microfluidics

Microfluidics emerge as an important tool for constructing various hydrogels structures (e.g., microfibers, microparticles, and hydrogel building blocks) with homogeneous size and controlled shape in the fields of tissue engineering and cell biology study. Hydrogel microfibers are normally fabricated after experiencing chemical or photo polymerizations on the laminar flow-based multiple phases coaxial flowing systems.^13^ As a paradigm, researchers used microfluidics to construct co-axial flow-based chitosan microfibers on which the harbored human hepatocarcinoma (HepG2) cells retained liver-specific functional characteristics (e.g., albumin and urea synthesis).^139^

Other important applications of microfluidics is to fabricate microgels, microdroplets, or microparticles, which also takes chemical and photo-induced cross-linking as the synthetic principle for tissue engineering and drug delivery.^140^ Microfluidics technology can finely tune the process of microdroplets’ birth via precisely designing microfluidic channels because the specific geometrical shapes and inputs of microfluidic channels can control the flow velocity of extruded hydrogels. Previously, sodium alginate is a commonly adopted material to fabricate hydrogel microparticles via the water-in-oil emulsion method.^141^ Moreover, photo-cross-linked polymers (e.g., PEGDA) that could be cross-linked with each other are also widely used material to yield microparticles in microfluidics via the continuous flow lithography technique.^142^

Cell-laden hydrogel building blocks could also be fabricated using microfabrication techniques via thermal-, chemical-, or photo-induced polymerization mechanisms. As for the thermal-induced cross-linked hydrogels (e.g., collagen, agarose, Matrigel, etc.), the micro-molding technique is usually applied. Typically, cell suspension was firstly mixed with agarose thoroughly at 40 °C, and then they were deposited into pre-prepared polydimethylsiloxane microchannels and polymerized into microscale hydrogel tissue architecture during the cooling process.^143^ Cuchiara and colleagues employed this method to prepare a photocrosslinkable PEGDA-based multi-layer microfluidic hydrogel system. This system was used to accommodate cells and study how the substance transports within hydrogels and what impacts the transport and exerts effects on cell behaviors. These results showed that the nutrition diffusion and cell viability depended on the distance from the perfusion channel, where the lowest cell viability was observed when cells were located at close to peripheral regions (600–1500 mm distance from microchannels).^144^

#### Injectable vs non-injectable hydrogels

Generally, hydrogels can be classified into injectable and non-injectable one. Injectable hydrogels are equipped with some prominent advantages compared to non-injectable ones. In detail, most non-injectable hydrogels need to be implanted, while injectable hydrogels can be delivered to the disease sites in the minimally invasive injection manner. Notably, injectable hydrogel refers to those flowable materials which can pass through medial syringe needle and form an integrated bulk hydrogel subcutaneously or at muscle tissue. Specifically, the flowable materials can be further divided into two subgroups, namely hydrogel precursor or self-healable hydrogel. The former one is the classic injectable hydrogel, which can be gelatinized under different physiological stimulations (e.g., temperature, pH, light, redox, etc.) in vivo. For instance, the fibrogenesis process (phase transition) of collagen or Matrigel will occur under physiological condition (37 °C, pH = 7.4), which is beneficial for injection.^72^ Moreover, researchers have reported a Ce6-CAT/PEGDA hydrogel for tumor inhibition, which could be gelatinized in situ under 660 nm irradiation via generating free radicals, enabling robust photodynamic-immunotherapy by multiple stimulations.^9^

Self-healable hydrogels refer to those materials that are formed via dynamic chemical bonds like Schiff base and recover its network after damage. In particular, injectable hydrogels feature high flowability due to the motion of polymer chains and the dynamic variation of cross-linking bonds. Hence, under shear stresses, hydrogels can be easily injected and then their morphology/mechanical properties at targeted disease site are recovered. Very recently, different types of self-healable hydrogels have been reported, and they significantly broaden the application window of hydrogels. Typically, self-healable hydrogels show great potential in promoting blood vessel regeneration via inducing contractility-mediated integrin β1 clustering of human endothelial colony-forming cells (hECFCs) and promoting FAK activation and metalloproteinase expression.^145^

## Influences of hydrogel properties on cell behavior and signaling pathways

Accumulative evidences have indicated that cells could perceive and respond to their surrounding matrix. In turn, the physicochemical properties of ECM could affect the biological events of cells continuously.^146–148^ It is found that the physical properties of hydrogels, e.g., stiffness, pore size, viscoelasticity, architecture, degradability, etc., could modulate cell biology via altering mechanotransduction signaling.^149–151^ Moreover, the chemical properties of hydrogels associated with cell attachment sites, chirality, hypoxia-inducible functional groups, (ir)reversible cross-linking sites, etc., could modulate cellular integrin clustering-involved signaling cascades, which further determined cell fate.^41,152^ Moreover, the stimulus-responsive property of hydrogels (i.e., smart hydrogel) could also dynamically regulate cell biology, promising for a wide application domain.^153–155^ Herein, more details on the interactions between ECM (i.e., hydrogel scaffolds) and cells as well as the underlying signaling pathways are discussed.

### Physicochemical properties of static hydrogels for regulating cell biology

#### Stiffness

It is reported that the elastic modulus of the brain, muscle, and bone is 1,^156^ 10,^100,157^ and 100 kPa,^100^ respectively. However, in diseased tissue like tumors, the stiffness was significantly varied,^72^ which is believed to take the responsibility for tumor progression. Hydrogels can provide a certain scaffold structure that mimics the stroma matrix of cell survival for supporting harbored cells’ activities, which determines that the characteristic stiffness of hydrogel scaffolds should satisfy the physiological demands in a cell/tissue type-dependent manner. On this account, the stiffness of hydrogels should be cautiously chosen for specific applications when using hydrogel scaffolds as the tissue matrix substitute.

Astonishingly, hydrogel stiffness has also been demonstrated to affect cell activities and functions, such as cellular morphology,^158^ proliferation,^159^ migration,^160^ differentiation,^161^ stemness,^162^ etc. Hence, it is necessary to comprehensively understand how cells sense and respond to hydrogel stiffness, which could navigate the future design of hydrogels. Basically, the mechanosensors that can realize the sensing and responses to hydrogel stiffness variation cover cellular integrins,^163,164^ focal adhesion kinases (FAKs),^165^ Rho GTPases,^166^ cellular stress fibers,^167,168^ etc. The cells would detect the substrate stiffness and then accordingly modulate the FA complexes and cytoskeleton contractility based on the requirements of varied cell-ECM adhesion strengths, eventually achieving intra- and extra-cellular force homeostasis.^169^

**Fig. 3:**
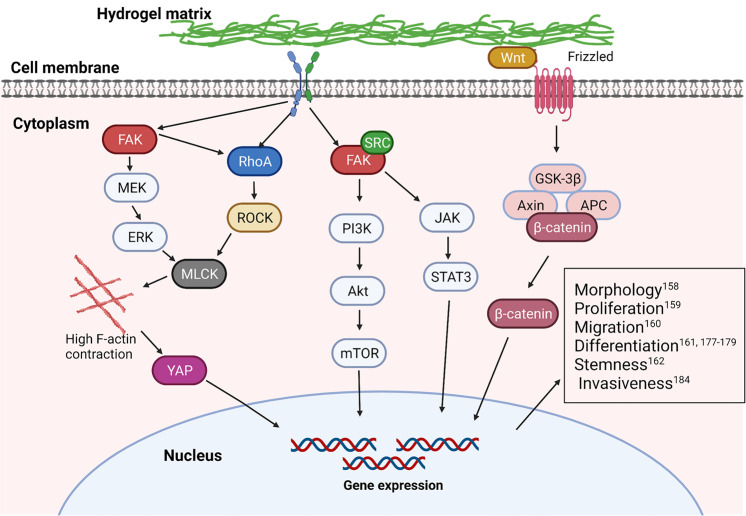
The outlined image for indicating the representative cellular mechanosignaling pathways induced by varied hydrogel stiffness. Hydrogel stiffness was demonstrated to correlate with many activations of focal adhesion kinase (FAK) signaling, RhoA signaling, and Wnt signaling and simultaneously regulate cell morphology, proliferation, migration, invasiveness, differentiation, and stemness.^417,418^ The figure is made with biorender (https://biorender.com/)

The stiffness-induced canonical mechanotransduction signaling pathways contain integrin-dependent FAK signaling,^170–172^ Rho/ROCK signaling,^173,174^ YAP/TAZ signaling,^26,175^ Wnt/β-catenin signaling,^176^ etc., all of which are responsible for the conversion of mechanical forces into biochemical signals and determine the terminal cell fate (Fig. 3). Several groups demonstrated that stem cells and cardiac fibroblasts differentiation are associated with substrate stiffness, which is believed to be a mechano-regulation process.^177–179^ Specifically, stem cells prefer to be differentiated into osteocytes when cultured on rigid polyacrylamide hydrogel substrate with stiffness (Young’s modulus, *E*) at approximately 42.1 kPa in comparison to soft one with stiffness at around 7 kPa.^178^ As well, another independent study showed that soft polyacrylamide hydrogel matrix (*E*, ~1 kPa) benefited stem cell differentiations into those cells enriched in chondrogenic and adipogenic lineage features. Once cultured on the rigid matrix (*E*, 15 kPa), stem cells tended to differentiate toward smooth muscle cell lineage. Notably, stem cells cultured on a rigid substrate displayed greater spreading, produced more stress fibers, and harvested a higher proliferation rate.^177^ Moreover, rigid gelatin hydrogel substrate (storage modulus, *G*’ = 2280 Pa) was reported to promote cardiac fibroblast differentiation into myofibroblasts with greater spreading, suggesting cardiac fibrosis compared to the soft one (*G*’ = 90 Pa).^179^ These cases also highlighted that the mechano-regulated cellular phenotypic preferences under different stiffness stimuli were potentially associated with FAK/(extracellular signal-regulated kinases) ERK signaling. In detail, the activated FAK could target paxillin, which would further activate mitogen-activated protein kinase (MEK), ERK and myosin light chain kinase (MLCK). Finally, the increased MLCK promoted cellular actin-myosin expression and myogenic cell differentiation on the rigid substrate.

Inspiringly, another molecular signaling transduction pathway was activated by substrate stiffness to drive the specific cell differentiation into the myogenic lineage. It has been demonstrated that mechanosensor (i.e., RhoA) could respond to external substrate stiffness, where the rigid substrate could activate RhoA/Rho-associated protein kinase (ROCK) signaling. Subsequently, the ROCK signaling activation could spontaneously activate MLCK, and further drive stem cell differentiation into myogenic lineage. Moreover, YAP/TAZ could also perform as mechanosensors and mechanotransducers to sense and respond to external matrix stiffness, which usually acted as the classic signaling pathway to regulate cell proliferation and fate, tissue regeneration and tumorigenesis.^180–183^ Related researches showed a cytoplasm-to-nucleus shifting of YAP/TAZ when the ECM stiffness increased, which resulted in the facilitated fibroblast proliferation.^181^ In contrast, the activated intranuclear YAP/TAZ could promote hMSCs differentiations toward osteogenesis in the absence of proliferation via interacting with β-catenin upon exposure to a rigid substrate.^183^ In another independent study, researchers demonstrated that MMP-7 expression in human colorectal cancer cells was upregulated via activating YAP, MRLC, and EGFR when cultured on rigid polyacrylamide substrate (*E*, 126 kPa) and consequently resulted in the poor prognosis in comparison to that cultured on the compliant substrate (*E*, 2 kPa).^184^ Notably, the matrix stiffness could also regulate the Wnt/β-catenin signaling pathway to control cell behaviors. In this regard, it is found that higher matrix stiffness could induce β-catenin accumulation and translocation into cell nuclei, followed by binding to T cell factor/LEF co-activators, during which chondrocytes de-differentiation with increased collagen I and β-catenin expression levels as well as decreased collagen II expression levels was accompanied.^176^ More intriguingly, matrix stiffness was found to potentially regulate transforming growth factor (TGF)-β^185^ and bone morphogenetic protein (BMP)^186^ receptor expression and spatial organization, integrin subunits (e.g., α1, β1, αVβ3, and β3) expression^23,187^ and intracellular reactive oxygen species level,^188^ consequently affecting related signaling pathways and cell functional characteristics.

It is worth noting that cell responses and actions to matrix stiffness in 2D and 3D hydrogel-based ECMs are different,^167,189,190^ and cell spreading in 3D situation was contrary to that on 2D substrates. In detail, cells spread across a large area on rigid collagen-based hydrogels, but were shaped into a round pattern on a soft one in the 2D context. In contrast, in the 3D setting, cells were gathered into round morphology within the rigid collagen-based hydrogels, but displayed a spread shape within soft one.^191^ Besides the differences in cellular distribution between 2D and 3D hydrogel-based ECMs, the cytoskeletal structure at the molecular level was also different. There were thick bundles of well-developed ventral stress fiber in 2D cell culture, while only a few thin stress fibers were found in cells’ cortex within a 3D setting.^167^ Also, the cell signaling between two ECMs also differed, where the cell adhesion in 2D ECM was determined by the integrin-based FAs that occurred at the cell–substrate interface. As a comparison, besides few FAs at the interfaces between cell-matrix, the majority of cells in a 3D context preferred to yield cell–cell contacts like cadherins. These differences are attributed to that 3D culture scaffolds provide 360° unrestricted interactions as compared to the pre-determined apical-basal polarity in 2D culture.^192^ Overall, matrix stiffness is a critical determinant for cell fate. From the standpoint of material science, rational design of hydrogel scaffold with physiologically matched stiffness as ECM surrogates is pivotal for directly acquiring proper cell functions, which will be also beneficial for the studies of tissue engineering and cancer therapy.

To make the stiffness of hydrogel scaffolds meet the demands of practical biomedical application, many methods have been developed to modulate the stiffness of hydrogels.^3,37,45^ Lots of experiences indicate that nanoparticles doping^193–195^ or chemical cross-linking^7,196,197^ are two dominant methods for enhancing mechanical properties. Nevertheless, stiffness is not the sole cause capable of determining cell behaviors in the 3D context, and some other parameters, such as matrix pore size, viscoelasticity, etc., could also exert roust influences on cell biology simultaneously.

Besides stiffness level, it has been accepted that substrate stiffening time also influence cell biology, e.g., regulating hMSCs lineage commitment. Briefly, adipogenic differentiation emerged only at the stage of late stiffening, whereas osteogenic differentiation could be observed at early substrate stiffening. However, these distinct cell responses arising from the alteration of substrate stiffing time has not been observed in static substrate with identical stiffness. This unexpected result indicated that the complex interplay of time-dependent stiffness signaling for regulating cell biology could be utilized to predict tissue development, wound healing process, and disease progression.^37^ As well, the stiffing time matters for stem cell differentiation, fibroblasts activation and cancer cell invasion, which furnishes important basis for tissue regeneration and drug development. Overall, the dynamical regulation of hydrogel physiochemical properties for recapitulating dynamic microenvironmental characteristics in vivo deserves to be taken into serious consideration, which is really beneficial to harness our understanding of cell biology.

#### Pore size

Porous ECM network could impose physical spatial confinement effects in varying degrees on dwelled/traveling cells via changing pore sizes, and further influence individual cell behavior and multicellular organization.^198–200^ For example, cancer cells could overcome the confinement effect of the primary tumor matrix with 1–30 μm pores and travel to a distant location.^201,202^ Both cancer cell migration and stem cell homing would inevitably experience the intravasation and extravasation processes to travel across blood vessels, during which cells would pass through the 1–2 μm gap between ECs via squeezing their own cell body.^203,204^ Porous structure with different pore sizes is another important physical characteristic of hydrogel scaffolds. It is believed that these pores could also affect cell activities. The inconstant porous structure can regulate many physiological activities and decide the success or failure of embedded cells or drugs for various lesion applications since the porous channels serve as the transporting passages of nutrient, metabolites and other substances. Hence, a systematic and deep understanding of the influences of hydrogel pore size-induced cell confinement on cell biology is favorable for improving regenerative medicine and cancer therapies.

**Fig. 4:**
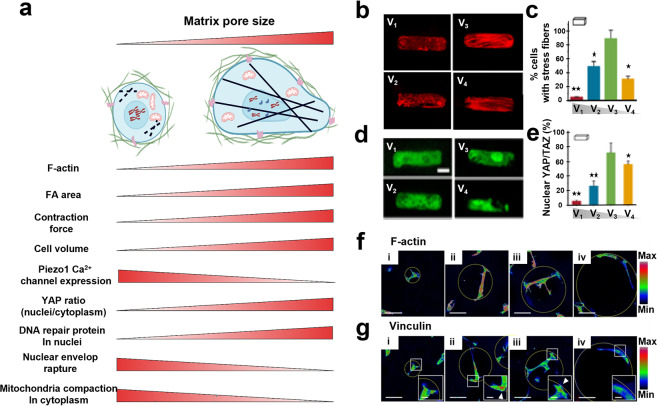
The influences of the pore size of hydrogel scaffolds on cell biology. **a** Influence summary of pore size on cellular compartment, molecular function, cytoskeleton arrangement, etc. The figure is made with biorender (https://biorender.com/). **b**–**e** Pore size (volume, V_1_ > V_2_ > V_3_ > V_4_)-dependent cell stress fiber formation (**b**, **c**) and YAP cytoplasm–nuclei translocation (**d**, **e**). *p < 0.05, **p < 0.01. Reproduced with permission.^25^ Copyright 2015, Springer nature. **f**, **g** A case focusing on how pore size (pore diameters: 47.0 ± 2.2 μm (Group i), 84.8 ± 11.0 μm (Group ii), 147.9 ± 7.2 μm (Group iii), and 198.7 ± 9.1 μm (Group iv)) affected F-actin (**f**) and Vinculin (**g**) expression in mesenchymal stromal cells. Reproduced with permission.^212^ Copyright 2016, Springer nature

At the molecular level (Fig. 4a), the cell confinement within hydrogels would influence cell membrane protein-regulated force transmission,^205^ cytoskeleton rearrangement,^25,206^ organelle distribution,^207^ nuclear membrane protein, and chromatin reorganization^208,209^ via activating various mechanotransduction signalings, and all these alterations will finally affect cell morphology,^24^ migration,^210^ invasion,^211^ differentiation,^212^ etc. For instance, the space confinement within collagen hydrogel (pore size <1 μm) could result in the diffusions and distributions of FA proteins (e.g., vinculin, paxillin, talin, α-actinin, zyxin, VASP, FAK, and p130Cas) throughout human fibrosarcoma cell cytoplasm rather than cell membrane where FA aggregates were found to routinely distribute. Intriguingly, those diffused proteins still reserved the ability to modulate cell migration via affecting protrusion activity and matrix deformation.^213^ Moreover, it is observed that the spatial confinement could lead to the retention of mechanosensitive protein YAP/TAZ in the cell cytoplasm, and this process was probably regulated by Rho GTPase and actin cytoskeleton tension.^214,215^ In Fig. 4b–e, hMSCs were confined within methylated HA hydrogel wells with different volumes (V_1_ > V_2_ > V_3_ > V_4_), wherein a significantly elevated stress fiber expression was observed in cells incubated within V_3_ wells (Fig. 4b, c). Meanwhile, high YAP/TAZ expression and evident cytoplasm-nuclei translocation were observed in V_3_ group compared to the rest (Fig. 4d, e). Concurrently, the well-volume difference-induced confinement effect resulted in different cell differentiation preferences. Typically, the V_3_-induced confinement propelled the transformation of stem cells towards osteogenesis featuring more ALP expression in comparison to V_1_.^25^ Additionally, the confinement was confirmed to upregulate CXCR2 chemokine receptor expression and heighten the intracellular calcium level, and both of which had close correlations with cytoskeletal remodeling and cell contractility.^216^ Protein kinase C has been also documented to participate in cellular cytoskeletal reorganization in the presence of confinement, and the marriage of kinase C inhibition and retinoic acid could cooperatively retard cell migration.^217^

Hydrogel scaffold-incurred confinement could also affect the nuclei composition, where it could lead to DNA damages^218^ and disrupt cell division.^219^ As an important mechanosensor, cellular nuclei remodeling will occur when the environmental pore radius is <7 µm.^218^ As a paradigm, the confinement (7–9 μm) leaded to the prolonged mitosis time (~2 times) of retinal pigment epithelial (RPE1) cells in comparison to the unconfined cells. Moreover, in the presence of confinement condition, chromosome segregation errors (65%) in HeLa cells and micronuclei (25%) in RPE1 cells were observed obviously.^220^ Also, the mobile proteins associated with DNA repair or nucleases were accordingly decreased in nuclei when the confinement degree rose. Especially, only chromatin could be maintained inside nuclei when the pore size was decreased to 2 µm,^221^ and the variation dynamics of chromatin was found to be regulated by both cytoskeleton and nucleoskeleton. Simultaneously, confined cells could give rise to fewer laminA/C and more dynamic heterochromatin foci, while an opposite trend would be observed as cells exhibited polarized morphology.^222,223^

Apart from affecting cellular compartment, cell biological activities were also significantly disturbed by the pore size of hydrogel scaffolds. For example, the division and proliferation of osteosarcoma cells could be significantly inhibited when confined in glass tubes with 8 µm diameter.^219^ McAndrew et al. demonstrated that stem cells preferred to differentiate into osteogenic lineage when cultured within gelatin-based scaffolds with smaller pore volume (30 μm^2^) than the larger one (100 μm^2^).^224^ Another independent study also confirmed that the pore size of gelatin scaffold could modulate stem cell differentiation via regulating intracellular actin cytoskeleton organization and FA (e.g., α2 and α5 integrins) distribution on cell membrane (Fig. 4f, g).^212^ Moreover, the confinement arising from hydrogel matrix pores also dictated the migration mode of cancer cells. Researchers reported that cancer cells would switch their migration pathway from mesenchymal to ameboid-like migration ways when they were entrapped within hydrogels. This transition was believed to be associated with RhoA signaling, and this transition tremendously reduced Rac1 activity.^225^ Moreover, cancer cells could dynamically and alternately adapt their metabolism to confinement and non-confinement during their collective migration process.^226,227^ Collectively, the pore size of hydrogel scaffold is an important indicator or hallmarker for cell biology and fate, and more attention needs to be paid when preparing hydrogel scaffolds objective to some certain application.

#### Viscoelasticity

**Fig. 5:**
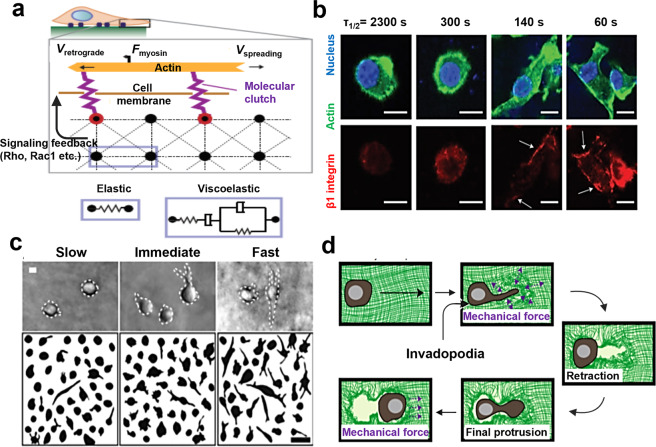
Clarifications and evidences on how hydrogel viscoelasticity regulated cell biology. **a** Schematic image of the interaction between cells and elastic/viscoelastic hydrogel via activating Rho and Rac1 signaling pathways. Reproduced with permission.^14^ Copyright 2015, Springer nature. **b** A case depicting how hydrogel with fast stress relaxation promoted stem cell spreading and β1 expression and led to integrin clustering. Reproduced with permission.^27^ Copyright 2015, Springer nature. **c** Protease-independent invasion way in cancer cells when cultured in viscoelastic hydrogels with slow, immediate, and fast relaxation rates, and **d** schematic on the migration mode of cancer cells via progressively widening surrounding hydrogel matrix with invadopodia rather than proteases. Reproduced with permission.^240^ Copyright 2018, Springer nature

It is extensively accepted that tissues were characterized to possess viscoelasticity property.^228–230^ Moreover, hydrogel biomaterials including ECM-derived components (e.g., collagen, fibronectin (FN), etc.) and non-ECM-derived materials (e.g., alginate, chitosan, etc.), also show the viscoelastic properties represented by stress relaxation or creep behavior.^1,231^ These viscoelastic properties regulated the interactions of harbored cells with surrounding matrix, and could elicit the differences in cell spreading,^14^ proliferation^232^ and differentiation^27,233^ in comparison to those cells without entrapment by hydrogel scaffold matrix. Therefore, viscoelastic properties modulation is also of great importance for acquiring satisfactory hydrogel matrix objective to a desired application. There are many influencing factors that get command of hydrogel viscoelasticity, e.g., precursor composition and concentration,^234^ MW,^235^ chain flexibility,^236^ cross-linking density or method (e.g., dynamic cross-linking bonds),^145,237^ etc. As cells were placed onto viscoelastic hydrogel substrates, cells’ traction force was dynamically changed over time via Rho and Rac signaling regulation (Fig. 5a). At the beginning, the traction force and strain force produced from cell motion and spreading or deformation on the surrounding hydrogel matrix would be resisted due to the rigidity of hydrogel scaffolds. As time elapsed, these forces were gradually decreased due to the counteraction effects caused by various dissipative events such as the unbinding of weak bonds, polymer disentanglement, protein unfolding, and molecule slipping within the hydrogel.^238,239^ For instance, alginate has been extensively used as the skeleton material to tune the viscoelasticity (e.g., stress relaxation speed or plasticity) of as-prepared hydrogels via varying its MW. The finely regulation of viscoelasticity is convenient for exclusively studying the effect of hydrogel stress relaxation on cell behavior because cells could not degrade alginate-based hydrogels.^27,240,241^ As well, the finely tuned viscoelastic property could affect cell behaviors independent of substrate stiffness.

Stress relaxation speed is a hallmark of viscoelasticity. Intriguingly, alginate with low MW (35 kDa) displayed a faster relaxation rate (170 +/− 20 s) compared to that with high MW (MW 280 kDa, 3300 +/− 800 s) even though both of them shared the approximately identical elastic modulus. This rapid relaxation rate was beneficial for cell contraction force induced-mechanical matrix remodeling, which also allowed increased RGD ligands clustering in the hydrogel and further facilitated enhanced cellular β1 integrin expression, FA formation and YAP nuclei translocation in cells (Fig. 5b). More interestingly, the fast relaxation rate of hydrogel scaffold matrix also promoted the spreading, proliferation and osteogenic differentiation potential of carried MSCs.^27^ Under the same hydrogel scaffold system mentioned above, researchers found that MDA-MB231 breast cancer cells in high plastic alginate based-hydrogel matrix could extend their invadopodia protrusions to mechanically open up micro-sized channels for boosting their migration rather than following the traditional protease-dependent migration (Fig. 5c, d).^240^ Studies also demonstrated that the fast relaxation of the RGD-free alginate matrix could significantly promote cartilage matrix formation and maintain cell phenotype with less interleukin (IL)-1β secretion. This result indicated that embedded cells could also sense cell volume confinement in an adhesion-independent mechanotransduction mechanism.^241^ Moreover, in another separate study, the mechanosensing protein, i.e., YAP, was translocated into skeletal muscle cell nuclei on the substrate with stress relaxation.^181,242^ More deeply, it is reported that the integrity of long intergenic non-coding (LINC) complexes were responsible for regulating intracellular tension via modulating actin cytoskeleton and formin FHOD1 and adapting cells to their surrounding soft matrix.^243^

However, it is critical to note that the stress relaxation that mediated the increased cell proliferation and spreading only occurred to matrices with lower stiffness because this effect is trivial and neglectable in comparison to the dominant stiffness effect in both 2D and 3D situations. As a evidence, cells spreading and proliferation were augmented when they were cultured on stiffer 2D hydrogel substrate compared to those cultured in the soft substrate featuring a high relaxation property.^28,239^ Taken all above together, the underlying mechanism of cell responses to stress relaxation-involved substrate is verified to depend on activations of cellular β1 integrin, actin polymerization and actomyosin-based contractility, YAP and LINC complexes, etc. In this regard, it is concluded that the substrate viscoelasticity could influence cell behaviors via activating mechanotransduction signaling pathways, akin to substrate stiffness. Collectively, the viscoelasticity property of hydrogel substrate is a critical design parameter for hydrogel fabrication.

#### Architecture

The architecture features (e.g., fibril diameter, fiber alignment) of the ECM network in vivo display dependent associations with tissue type and location and simultaneously determine how cells interact with their surroundings.^72,244^ For instance, the switch of collagen fibril architecture from thin and wavy morphology to thick and paralleled arrangement represents tissue fibrosis.^245,246^ Generally, hydrogel architecture was decided by the composition itself and the scaffold fabrication approach. As reported, the fiber diameter of synthetic scaffold ranged from nanofibers to microfibers because of the inherent biomaterial properties^5^ or fabrication process^247,248^, where the fiber alignment could be finely tuned from 0° to 180°. Hereby, it is concluded that hydrogel architecture can potentially influence cells’ activities and signaling cascades. The deep understanding on the underlying principle of hydrogel engineering architecture in manipulating cell biology behavior and signaling cascades will be helpful for the rational design of tissue-engineered scaffolds.

**Fig. 6:**
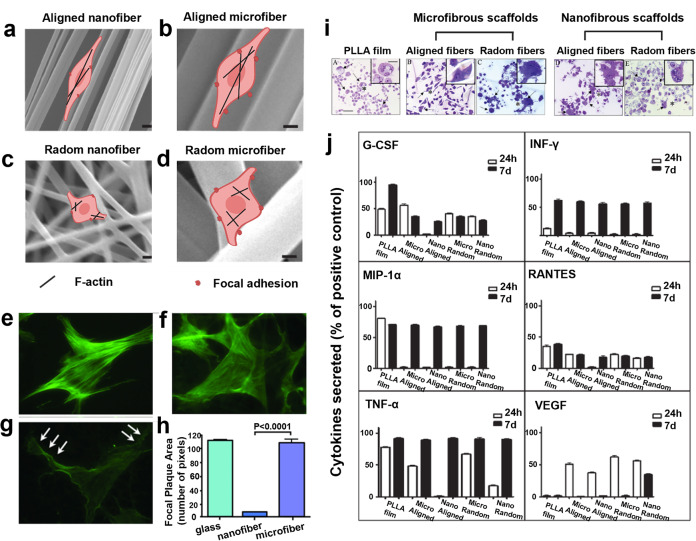
The effects of hydrogel architecture on cell activities. **a**–**d** Schematic image of the interaction between cell and hydrogel scaffolds with different topographies. Cells generally exhibited a spindle-shaped morphology on microfibers (**b**, **d**) or aligned fibers (**a**, **b**), while evolved into the rounded morphology on nanofibers or randomly oriented fibers (**c**). Reproduced with permission.^249,419^ Copyright 2013, Wiley-VCH. The figure is made with biorender (https://biorender.com/). **e**–**h** Fibroblast F-actin staining (green) on glass (**e**), microfiber (**f**), and nanofiber (**g**), as well as the quantification analysis of focal plaque area (**h**). The arrows represent membrane protrusions (“cork-screw” ruffles). Reproduced with permission.^30^ Copyright 2011, Wolters Kluwer Health, Inc. **i**, **j** Evidences on how the architecture of hydrogel scaffolds regulated macrophage morphology (**i**) and cytokine expression (**j**), where microfibers (1–50 μm) induced M1 microphage activations and more proinflammatory cytokine secretions compared to fibers with a diameter of 200–600 nm. Adapted with permission.^257^ Copyright 2011, American Chemical Society

Cell morphology is also decided by the hydrogel scaffold architecture. It is reported that cells could be evolved into the spindle-shaped morphology on microfibers or aligned fibers, but spontaneously transformed into rounded morphology on nanofibers or randomly oriented fibers (Fig. 6a–d).^248^ Moreover, the migration velocity of cells on nanofibers or aligned fibers was higher than that on microfibers or randomly oriented fibers.^249,250^ Specifically, massive FA complexes were observed when cells that were cultured on the substrate with large fiber diameter (1–4 μm), and actin polymerization was accompanied. This phenomenon further enhanced cell spreading, aspect ratio, alignment, and elongation.^248,251,252^ Other reports also confirm that aligned fibers could induce the upregulation of FA-related protein expression levels (e.g., vinculin and paxillin) in adhered cells (Fig. 6e–h).^30^ Excitingly, due to the different cellular responses to fiber features, gene expression and cell fate accordingly varied. To exemplify it, bovine chondrocytes were cultured on thin electrospun chitosan nanofibers (400 nm), and chondrogenic markers SOX5/9 and collagen II in these bovine chondrocytes were upregulated in comparison to those cells cultured on fibers with a diameter of 700 and 1.33 μm, suggesting the high pro-chondrocytes differentiation potential on fibers with thin thickness.^253^ Indeed, another study also evidenced that hMSCs on thin fibers preferred to differentiate into osteocytes, evidenced by the increased RUNX2 and osteocalcin expression levels in the presence of osteoinductive media when comparing nanofibers with 400 and 1400 nm thickness, respectively.^254^ Hsia et al. demonstrated that human fibroblast displayed more prominent actin stress fibers and larger FA complexes on microfiber scaffold (2.62 ± 0.39 µm) than those on nanofiber scaffold (0.66 ± 0.14 µm).^30^ In consideration of this phenomenon, it is highly possible that the slower migration speed of cells on microfiber resulted from the larger lamellipodia (actin network) of cell trail since large lamellipodia disable cells to slide forward on microfibers compared to that on nanofibers. Identical result is verified by other groups, where some researchers demonstrated that GBM cells migrated faster and expressed higher epithelial-to-mesenchymal transition (EMT) markers on aligned chitosan–PCL polyblend nanofibers compared to the corresponding microfibers. As for other stem cells, it is found that neural stem cells (NSCs) prefer to differentiate into neuronal cells cultured on aligned fibers in comparison to randomly oriented fiber scaffolds.^255,256^ Overall, the variable cell behaviors, intracellular actin and FA protein expression profiles indicate that fiber diameter and orientation-dependent cell biology also follow the mechano-regulation process.

Excitingly, fiber diameter and fiber alignment could induce different immune responses in different cell types.^257–260^ For instance, microfibers (1–50 μm) would induce M1 microphage activation and more proinflammatory cytokine secretions compared to fibers with a diameter of 200–600 nm (Fig. 6i, j).^257^ Moreover, fiber orientation is closely correlated to the responses of tendon fibroblasts (TFs) to paracrine signals stemming from activated macrophages or proinflammatory cytokines. Specifically, TFs would downregulate ECM protein-related gene expression and upregulate MMP gene expression on randomly oriented electrospun PCL scaffold compared to the aligned one, thus resulting in degenerative tendon diseases.^29^

Notably, the difference of cell biology induced by fiber diameter or orientation should be cautiously investigated because cells would be probably exposed to many fibers rather than only one at one moment. Typically, NIH3T3 fibroblasts lamellipodia would inevitably encounter other fibers when cultured on fibers with a diameter of 150 nm. Instead, these cells would extend along with one fiber when the fiber diameter was 750 nm.^252^ Additionally, fiber alignment could mediate cell proliferation, but the proliferation degree depended on cell type. For instance, human ligament fibroblast and ECs displayed neglectable proliferation difference on both aligned and randomly oriented fibers,^261,262^ while more proliferations of hMSCs and keratocytes were detected on aligned fiber.^263,264^ Moreover, corneal epithelial cells approximately failed to proliferate when they were cultured on non-aligned fibers.^264^ Collectively, the fiber parameter is an indispensable consideration factor when designing scaffold implants.

#### Degradation

**Fig. 7:**
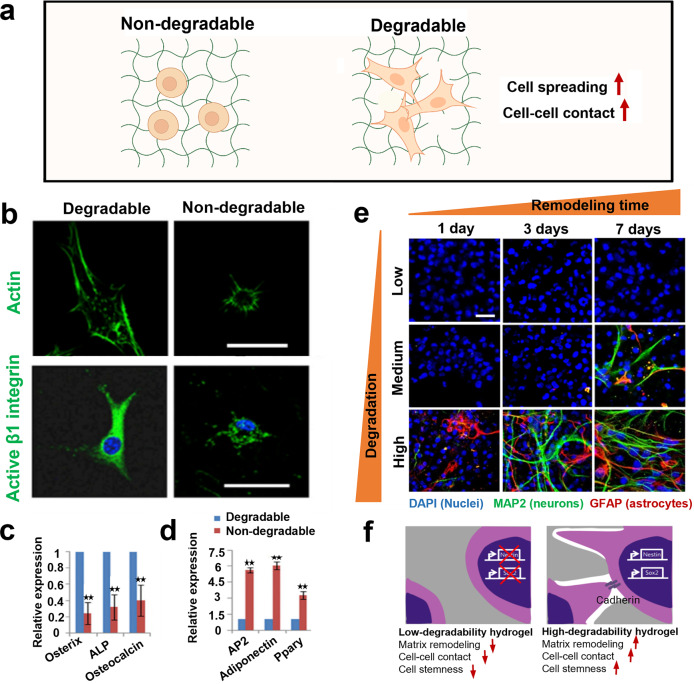
The influences of hydrogel degradability on cell biology. **a** Schematic image of the interaction between entrapped cell and degradable/non-degradable hydrogels. The figure is made with biorender (https://biorender.com/). **b**–**d** Explorations on how hydrogel degradability promoted stem cell spreading and β1 integrin activation within 3D PEG-based hydrogels (**b**) and induced distinctive differentiation preference (**c**, **d**), e.g., osteogenesis and adipogenesis within the degradable and non-degradable hydrogel, respectively. **p < 0.01. Reproduced with permission.^275^ Copyright 2013, Elsevier. **e**, **f** Tests indicating how hydrogel degradability enhanced neural stem cells’ stemness (**e**) via permitting cell–cell contact and inducing Nestin and Sox2 expression (**f**). Reproduced with permission.^17^ Copyright 2017, Springer nature

Apart from the above physical parameters, the stroma matrix degradation in vivo has been also validated to largely influence cells behaviors (e.g., cell spreading and cell–cell contact) and functional characteristics, including cancer cell invasiveness,^265,266^ multicell aggregation formation,^267^ and stem cell lineage commitment.^17,18,268^, In turn, the degradation behavior could be continuously and dynamically remodeled by the dwelling cells (Fig. 7a).^72^ Hence, the hydrogel degradability that can be mediated by enzyme catalysis, ester hydrolysis, or photolytic cleavage is considered as another important parameter for hydrogel design and its corresponding application.^1,231^ In skin tissue regeneration, the ideal degradation property needs to be taken into consideration to benefit cell proliferation and blood vessel infiltration and simultaneously balance inflammatory effect that are probably resulted from the side effects of degraded products.

It is well established that canonical cancer cell migration including EMT-mediated mesenchymal pattern or collective pattern required the proteolysis-dependent ECM degradation to leave paths for cell invasion.^265,266,269^ During this process, the cellular actin dynamics, integrin-based ECM adhesion (e.g., β1 and β3), and proteolytic ECM cleavage collaborated in an ordered and efficient manner.^270,271^ Moreover, protease expression would be increased due to the upregulation of LOX expression in tumor when the collagen cross-linking increased, which would lead to the enhanced MMP-dependent ECM cleavage and further enlarge the pore size of the matrix for supporting cancer cell migration.^272,273^ Meanwhile, invadopodia on the cell membrane become more stable via the enhanced integrin-mediated signaling due to the MMP-mediated births of matrix patterns and microtracks. As reported, the migration capacity of cancer cells was significantly impaired after treatment with a metalloproteinase inhibitor. As a paradigm, the silence of matrix proteolysis or ROCK could mute myosin II and Rac1-mediated protrusive activity of cancer cells within the collagen-based 3D matrix.^274^ Tang et al. have reported that β1-integrin activation was necessary for the membrane-anchored metalloproteinase MT1-MMP (Mmp14)-induced proteolysis of the surrounding matrix.^275^ Specifically, MT1-MMP+/+ SSC cells displayed a spreading morphology with activated β1-integrin and FAK in degradable PEG-based hydrogels compared to the non-degraded one (Fig. 7b–d). These data indicate that the matrix degradation-mediated varied cell behaviors are associated with the cellular mechanotransduction process. Moreover, multicell aggregation and tumor spheroids exhibited a bigger, less rounded and smooth morphology within degradable hydrogel in comparison to non-degradable ones.^276^

Hydrogel degradability was also validated to influence stem cell lineage commitment.^17,31,277^ With regulations by varied signaling pathways, skeletal stem cells would commit to osteoblastogenesis and adipogenesis in degradable and non-degradable PEG-based hydrogels (Fig. 7b–d), respectively, even though they were treated under the identical chemical factors (i.e., the co-existence of osteogenic and adipogenic factors).^275^ Moreover, another independent study also show that the cytoskeleton tension or engineered adhesive ligands availability displayed no association with hydrogel degradation-mediated neural progenitor cell (NPC) stemness maintenance in the absence of stimulated differentiation factors (Fig. 7e, f). Interestingly, the degradation demands relied on how matrix remodeling promoted cadherin-mediated cell–cell contact and initiated downstream β-catenin signaling.^17^ Moreover, the degradable hydrogel system could also promote stem cells to differentiate into chondrocytes in MMP-sensitive collagen-mimetic hydrogels or MMP-sensitive HA hydrogels.^268^ Additionally, Khetan et al. pointed out that the distinctive stem cell fate was modulated by degradation-specific traction stress. In their study, stem cells would differentiate into osteocytes when they were cultured in HA hydrogels that incorporated degradable peptides but transformed into adipocytes once embedded in non-degradable hydrogels through a delayed secondary cross-linking process. Specifically, they indicated that hydrogel degradation allowed cells to rearrange their cytoskeletal structure, resulting in a high degree of spreading and tractions. More importantly, cell fate affected by traction force was decided independent of cell morphology, because the upregulated cell tension was able to also induce osteogenesis even in the highly restrictive environment.^277^ In another independent study, researchers also showed that the fabricated PEG-based degradable hydrogel benefited stem cell proliferation and differentiation, thus holding high potential for bone engineering. Specifically, at the beginning, the degradable soft hydrogel that mimicked bone marrow contributed to stem cell spreading and proliferation and multipotency maintenance. Thereafter, when the cells migrated to the stiff surface that mimicked bone defects, cells could differentiate into osteoblast lineage.^15^ Depending on these unprecedented features, the degradable PEG-based hydrogels for culturing stem cells and regulating their proliferation and differentiation are expected to hold great potential in bone regenerative medicine.

#### Cell attachment sites

**Fig. 8:**
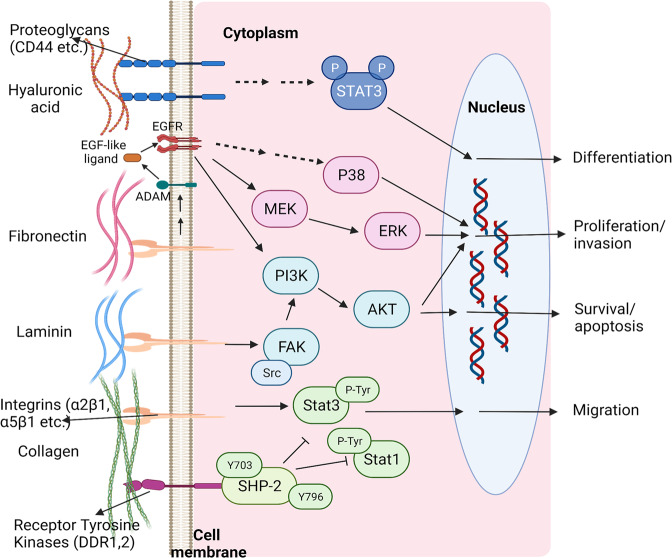
Different cell attachment site (chemical surface)-induced signaling pathways. Information is collected from published works.^34,279–281^ The figure is made with biorender (https://biorender.com/)

Cell adhesion in the surrounding matrix is determined by the cell adhesion molecules (CAMs) on the cell membrane. Summarily, CAM primarily includes integrins (e.g., α2β1, α2β1, etc.), proteoglycans (e.g., CD44), and receptor tyrosine kinases (e.g., DDR1,2), and these CAMs could specifically interact with certain ligands chelated in scaffold matrix.^278^ Therefore, the material used for hydrogel fabrication will exert potent influences on cell fate via regulating cell adhesion-induced signaling cascades. The exposed extracellular domain of different integrins permitted cells to specifically recognize ECM proteins typically such as FN,^279^ collagen,^280^ laminin,^281^, and other ECM components like HA, which, thereby, regulate cell adhesion,^41,282^ migration,^283^ differentiation,^284^ and apoptosis^285^ via varying signaling pathways (Fig. 8).

Generally speaking, cell attachment on the matrix is critically important for cell survival, and without appropriate attachment, the cells would potentially undergo anoikis.^285^ Hydrogels fabricated from ECM-derived proteins consisted of these cell attachment motifs, e.g., RGD motifs in collagen and FN,^276^ heparan sulfate-binding domains in FN.^283^ Varied fragments capable of resisting to protease binding on laminin could be specifically recognized by cellular integrins. However, myriad hydrogel materials, such as alginate,^286^ PEG-derivatives,^287^ etc., are bioinert. Hence, researchers always use RGD to modify the hydrogel polymers, so as to improve the biocompatibility of the hydrogel system and ensure cell viability. As a paradigm, Pinkse et al. demonstrated that b1-integrin-RGD peptides interaction favored integrin activation in isolated hepatocytes and resulted in ILK activation and pAKT phosphorylation, which could further protect cells from apoptosis.^288^ In another study, RGD peptide played a positive role in cardiac hypertrophic growth via S6K1 activation. Therein, RGD could interact with β3 integrin, followed by endocytosis and subsequent S6K1 activation in cardiomyocytes via regulating mTOR and MEK/ERK signaling pathways.^289^ Moreover, FN could trigger MEK/ERK and PI3K/Akt pathway activation via elevating integrin αv-mediated disintegrin and metalloproteases activity and eventually promote hepatocellular carcinoma (i.e., CBO140C12 cells) proliferation and invasion.^279^ As well, Zhang and co-workers reported a series of RADA16-I hydrogels, and these hydrogels were covalently conjugated with varied cell-adhesive ligands (e.g., RGDSP, TTSWSQ, and GFOGER) that were derived from FN, angiogenesis inducer CCN1, and type I collagen, respectively. Results showed that RGDSP-conjugated hydrogels could promote more stem cell proliferation, while TTSWSQ- and GFOGER-conjugated hydrogels could enhance osteogenesis with more calcium deposition when cultured in osteogenesis differentiation factor-contained media. This phenomenon was attributed to different ligands that could recognize and bind to different specific integrins and thus activate distinct signaling pathways.^290^ Notably, the laminin-integrin signaling is also beneficial for retinal ganglion cell (RGC) survival via beta1 integrin-FAK signaling activation especially after ischemia, indicating that the maintenance of homeostatic RGC–laminin interaction was promising for neuroprotection.^281^ Furthermore, the HA–collagen hybrid ECM has been documented to be able to trigger spontaneous M2-like polarity of monocyte/macrophage via CD44-mediated STAT3 activation in THP-1 cells. CD44 is a cell-surface glycoprotein antigen receptor^34^ and always upregulated in basal-like breast cancer tissue, suggesting the high risk of tumor metastasis.

It is worth to note that the density, spatial location, and mobility of ligands could also affect cell adhesion because FA complexes would disassemble when ligands were insufficient or sparsely distributed.^291^ As demonstrated by Maheshwari et al., fibroblasts exhibited a potentiated spreading behavior only when the RGD ligands were tethered within PEG-based substrate with an intercluster spacing of 300 nm.^292,293^ Collectively, it is vital to take consideration into which type, where, and how the ligands were conjugated within hydrogel scaffold during design process, which has profound implications for cell biology and targeted scaffold application.

#### Other hydrogel properties

**Fig. 9:**
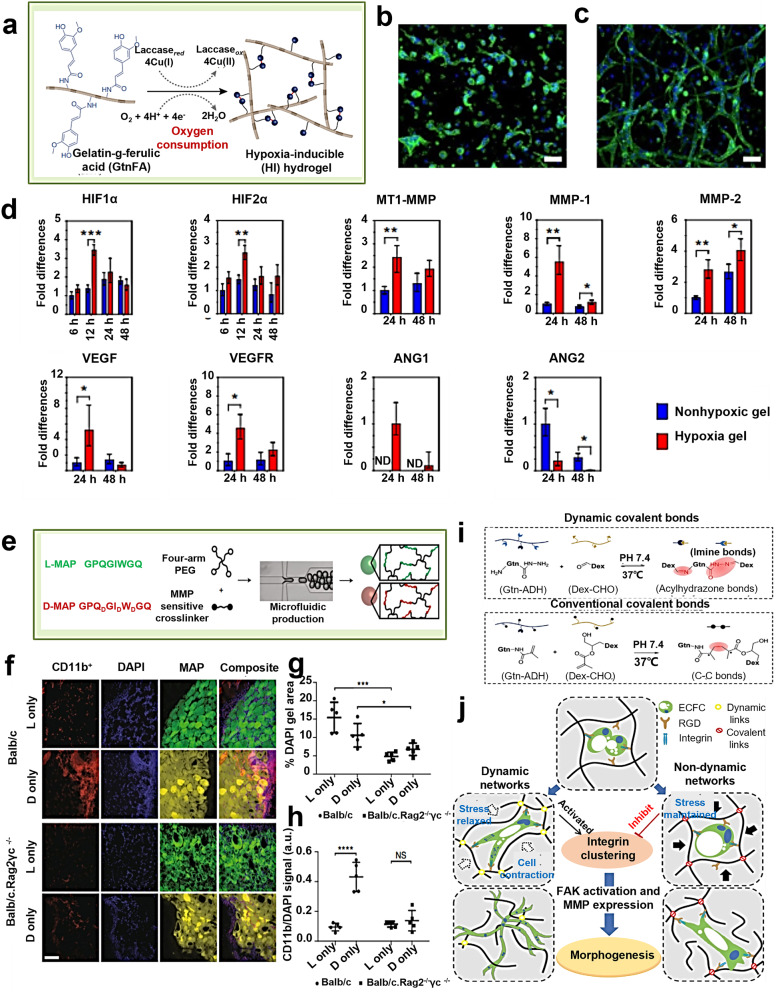
The effects of other hydrogel properties on cell biology and biomedical applications. **a**–**d** Hypoxia-inducible hydrogel design (**a**) could enhance blood vessel morphogenesis (**b**, **c**) with increased correlated gene expression levels (**d**). *p < 0.05, **p < 0.01 and ***p < 0.001. Reproduced with permission.^295^ Copyright 2014, Springer nature. **e**–**h** D- and L-chiral hydrogel preparation (**e**) and the induced immuno-responses (**f**–**h**), where hydrogels with D-chirality induced adaptive immune responses with CD11b+ myeloid cell recruitment compared to the L-chiral hydrogel in mice. (**g** *p = 0.0455, ***p = 0.0006; **h** ****p < 0.001). Reproduced with permission.^297^ Copyright 2020, Springer nature. **i**, **j** Self-healing hydrogel construction (**i**) and the enhanced angiogenesis via a series of signaling cascades, including integrin clustering, FAK activation, and MMP expression (**j**). Reproduced with permission.^145^ Copyright 2020, Elsevier

With the demanding rise of hydrogel-based biomedical application, more and more hydrogels with extraordinary properties emerged, such as O_2_-controllable hydrogels,^294,295^ chiral hydrogel,^296–298^ targeted proteins-loaded hydrogels,^287,299,300^ self-healing hydrogels,^145^ etc. Especially, the complex stroma microenvironment (e.g., hypoxia and low pH) inspired more hydrogels’ birth, such as hypoxia-inducible, O_2_-controllable hydrogels, etc. These strategies could regulate stem cell differentiation, cancer cell apoptosis, cell redox metabolism, and related signaling pathway.^295,301^ Park et al. developed a type of hypoxia-inducible ferulic acid-conjugated gelatin, and this gelatin holds great pro-angiogenesis potential of ECFCs by activating hypoxia-inducible factor (HIF) signaling cascades (Fig. 9a–d).^295^

The homochirality of amino acids and carbohydrates for influencing biology has been well explored, which provided us an insight into the necessity for hydrogel fabrication and application. Similar to amino acids and carbohydrates, hydrogel chirality could also determine cell biology, including cell adhesion, proliferation, migration, immune response, and gene expression, and present significant implications in tissue engineering. Researchers demonstrated that L-form chiral hydrogel could efficiently promote cell (e.g., stem cells, ECs, fibroblasts, etc.) adhesion, spreading and proliferation in comparison to D-form chiral hydrogel.^296,298,302^ The phenomenon was attributed to the augmented protein deposition on the L-form chiral hydrogel substrate because the association constant of FN on L-form chiral hydrogel was 0.1123 that was much higher than that on D-form one (0.0527). The intriguing stereo-specific interaction between chiral hydrogel and FN would further emit various signals to cells and result in different cell behaviors.^298^ Although D-form chiral hydrogels showed poor pro-cell activity, they were also equipped with some prominent advantages appropriate for certain applications, such as undifferentiated stem cell phenotype maintenance^296^ and adaptive immune response activation.^297^ In this field, Griffin et al. fabricated microporous annealed particle (MAP) scaffolds using d- or l-peptides for cross-linking. Compared to l-MAP hydrogel, d-MAP scaffold displayed a faster degradation rate in vivo, which would benefit IL-33+ type 2 myeloid cell recruitment and d-peptide targeted antigen-specific immunity activation. Furthermore, it would enhance cutaneous wound healing, accompanied with increased tensile strength and hair neogenesis (Fig. 9e–h).^297^

As far as we know, the exocrine growth factors/cytokines would be sequestered within the surrounding matrix, consequently affecting cell behaviors in an autocrine or paracrine manner. Given this, the presence of signal cues induced by hydrogels was also critical for application.^287,299,300^ Browne et al. developed a HA-based hydrogel decorated with high MW heparin for sequestering TGF beta1 growth factor. The modified hydrogels could regulate vascular network formation via TGF beta1/CD105 signaling modulation.^300^

Apart from above-mentioned properties, the cell contraction force and external applied force could do damages to hydrogel network structure, which tremendously limited hydrogel application. Inspiringly, the newly emerged self-healing hydrogels could recover its morphology and mechanical properties due to the reversible cross-linking repair after damages.^303,304^ It is believed that the combination of self-healing capacity with other properties during hydrogel design holds great potentials in tissue engineering and cancer therapy. Wei group compared the efficiency of vascular morphogenesis regulation between non-self-healing and self-healing hydrogels with identical stiffness (Fig. 9i, j). Results showed that the dynamic self-healing gelatin-based hydrogel could promote contractility-mediated integrin β1 clustering of hECFCs and result in FAK activation and metalloproteinase expression, which eventually enhanced angiogenesis in vitro and in vivo.^145^

Moreover, tissue-mimicking electroactive hydrogel materials are promising for various biomedical applications. In particular, when combining with the electricity-sensitive cells and tissues (e.g., skeletal muscle cells, nerve cells, etc.) that could be stimulated with external electric signals and then exhibited different cellular biological behaviors, more functions are accessible. For instance, researchers have incorporated tannic acid into polypyrrole hydrogel to promote NSC differentiation into neurons, accompanied with which the commitment into astrocytes was suppressed.^305^ Moreover, different hydrogel systems with photothermal effect have been reported, and they could result in cell deaths via destroying intracellular proteins and DNA under irradiations, laying a solid foundation to photothermal therapy (PTT) in clinics.^306^ As a typical paradigm, 3D biofunctional scaffolds composed of alginate and polydopamine (PDA) have been demonstrated with excellent abilities to suppress breast tumor growth and further guide tissue repair due to their good adherence affinities to adjacent healthy tissue.^307^

### External stimuli-responsive hydrogels for regulating cell biology

**Fig. 10:**
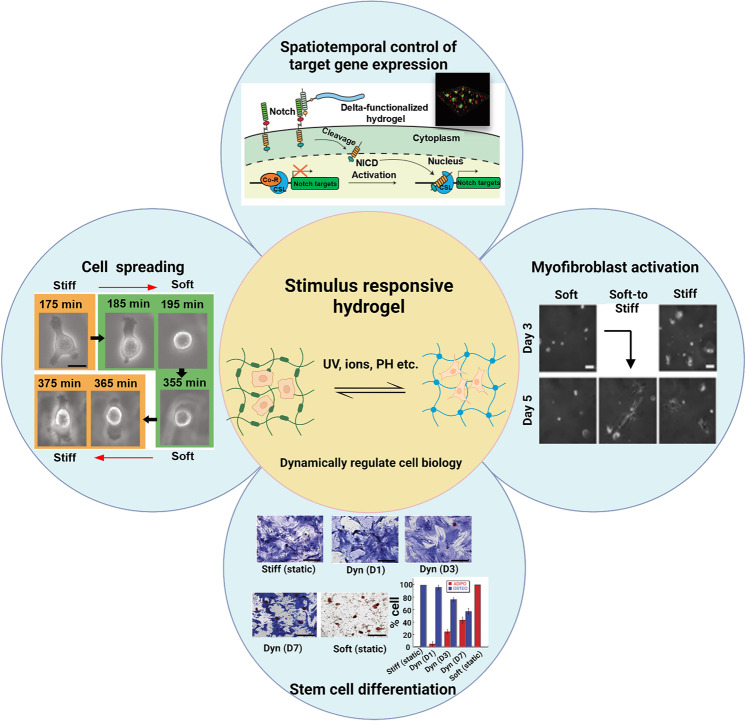
Influence recapitulation of stimulus-responsive hydrogels on dynamic cell microenvironment, spatiotemporal cell spreading control,^318^ target gene expression,^322^ myofibroblast activation,^35^ and stem cell differentiation.^37^ Adapted with permission from ref. ^318^ Copyright 2014, American Chemical Society. Reproduced with permission.^322^ Copyright 2015, Springer nature. Reproduced with permission.^35^ Copyright 2016, Springer nature. Reproduced with permission.^37^ Copyright 2012, Springer nature

Hydrogel systems with specific responses to an external stimulus (e.g., light, electric current, pH, temperature, ionic strength, etc.) are partially designed for reversible and irreversible on-demand modulation of biochemical and mechanical cues with spatiotemporal precision. They are available for real-time manipulation of cell microenvironment so as to mediate cell behaviors and functions in 2D and 3D contexts (Fig. 10).^308,309^ Additionally, the external stimulus-responsive hydrogels have been also extensively used as targeted drug delivery systems, e.g., pH/acid-responsive hydrogels for tumor therapy and glucose-responsive hydrogels for insulin delivery.^310^ In this section, we focused on the cell responses to the various smart hydrogel systems. A majority of studies that discussed smart hydrogel design paid more attentions to polymer modification, but their cell studies merely served as a proof of concept. Hence, more cell macroscopic responses should be discussed, and related signaling pathways should be elucidated. Enlightened by aforementioned discussions that stiffness, pore size, viscoelasticity, architecture, degradation, and cell adhesion sites in hydrogels could regulate cell behaviors, smart hydrogels after rational design are also expected to finely and dynamically tune these cell behaviors, such as cell adhesion,^311,312^ migration,^313,314^ differentiation,^315,316^ invasiveness,^317^ etc. Great advances have been made in smart 2D interactive hydrogel scaffolds for studying the influences of stimulus-responsive properties on cell biology. For instance, Desseaux and Klok fabricated a type of thermo-responsive RGD-containing thin film to regulate cell attachment and de-attachment on demand, where the dynamically temperature-dependent RGD availability on the film was achieved via surface-initiated atom transfer radical polymerization of HEMA, PEG methacrylate, and 2-(2-methoxyethoxy)ethyl methacrylate. Results showed that 3T3 fibroblasts could adhere on the film surface at 37 °C due to copolymer collapse-induced RGD presence but would de-attach at 23 °C because of RGD ligand disappearence.^318^

Wegner and co-workers reported another approach for dynamically controlling cell adhesion, called as the photo-cleavage method. In brief, the substrate was decorated with a photocleavable nitrobenzyl linker via click reaction. At the other end of the linker, NHS moiety could allow biomolecules’ (e.g., RGD) conjugation for promoting cell adherence. When light was applied, the conjugated biomolecules would be released, resulting in cell de-attachment.^319^ Similarly, in a 3D setting, the photo-responsive hydrogels also behaved as one of the most popular approaches for dynamically regulating the presence and removal of biological cues (e.g., RGD ligands) in a spatiotemporal-controlled manner.^320,321^ DeForest et al. developed a novel approach to selectively pattern protein vitronectin present within PEG-based 3D hydrogel, which, thereby, could spatially control the reversible differentiation of human MSCs (hMSCs) into osteoblasts with target gene expression. Especially, the photodeprotection-oxime-ligation sequence and ortho-nitrobenzyl ester photo-cleavage reaction within the hydrogel could bring about protein anchorage and removal, respectively.^322^

Besides chemical cues, the biomechanical properties could also be modulated dynamically for manipulating cell behaviors.^323,324^ Gillette et al. have reported an interpenetrating dynamic polymer network based on collagen-alginate (CoAl), wherein the alginate network as the second network was interlaced into the dominant collagen framework to tune the mechanics of hydrogel composite via inotropic gelation. Notably, the alginate network could be selectively de-cross-linked using a chelator-like sodium citrate, during which the influence on the network integrity could be overlooked. Correspondingly, the morphology of mouse fibroblasts switched from rounded morphology within the cross-linked composite hydrogel into a spreading state when the alginate network was eliminated. This result indicated that the physical confinement was another factor that could determine cell spreading.^325,326^ In another study, apart from the morphology difference, alginate detachment from CoAl composite hydrogel also triggered mechanics variation of hydrogels, which further led to the reversible phenotypic switch of inhabited cancer-related fibroblasts between inflammatory state (i-state, α-SMA^low^IL-6^high^) and myofibroblastic (m-state, α-SMA^high^IL-6^low^) state in cancer-associated fibroblasts.^327^ Moreover, the reactive oxygen species (ROS)-HIF1α mechanotransduction signaling axis was recognized as the underlying principle and take responsibility for the phenotypic alteration. To comprehensively understand the influences of biomechanical property variation of hydrogels on cell biology, Caliari et al. fabricated a methacrylated hyaluronic acid (MeHA)-based platform whose stiffening would be sequentially varied under visible light irradiation, which was used to study hepatic stellate cell mechano-regulated cellular behaviors during myofibroblasts activation. Interestingly, compared to the initial stiffened hydrogel, the sequentially stiffened hydrogel could foster myofibroblast differentiation via accelerating signaling kinetics of both early YAP/TAZ and late α-SMA markers.^35^ In another independent survey where a similar hydrogel stiffing method was adopted, it is also reported that the dynamic stiffening of MeHA hydrogels could modulate malignant transformation of mammary breast epithelial cells (i.e., MCF10A) via regulating paracrine and mechanical signaling. In particular, the tumor spheroids produced on the initial soft substrate would spread out and display EMT-like cell characteristics due to the activated transcription factor (e.g., Twist1, TGFβ, and YAP) when the substrate stiffing was carried on for 2 days. However, the spheroids state would be reversibly regained when the substrate stiffing was conducted for 10 days, indicating that the effects of cellular paracrine signals within the tumor on tumor metastasis overrode the external mechanical cues-induced ones.^36^

In light of the fact that the degree and time of stiffness closely correlated with cell biology, the external stimuli-responsive hydrogel that could be stiffened on-demand at any time allow researchers to study the cells response over time relative to stiffness changing. It is preferable for addressing the concern that conventional static hydrogel system only allows us to study cell behavior at the two end points of hydrogel stiffness.

## In vitro and in vivo potential applications of hydrogel

Due to the bio-mimicking properties of hydrogels, they have been extensively used for various biomedical applications as shown in Fig. 1, such as diseases model establishment, various cell culturing and delivery for tissue engineering and cancer therapy, smart drug carrier, bioimaging and biosensor, wearable/implantable devices, etc.

### 3D disease models for in vitro high-throughput drug screening

Currently, a majority of drug discovery and screening tests have been carried out on cells seeded on 2D plastic surfaces that were optimized for tissue culture. However, 2D culture failed to fully reflect the complex cell–cell paracrine crosstalk and cell-matrix interactions in vivo, resulting in the state of art that the drugs tested with high efficacy in lab always cannot meet the expectations in clinics.^328^ In addition, animal models also encountered some challenges such as high cost and large immune system difference far from the human being.^329^ More cost-effective and physiologically related bio-mimetic 3D hydrogels that serve as in vitro disease models have shown unprecedented advantages for high-throughput drug screening because hydrogels are able to mimic physiochemical properties of native ECM and provide optimal 3D cell growth environments. Moreover, abundant successful cases in vitro such as tumor model,^44^ tissue fibrosis models,^20^ corneal disease model,^45^ nerve disease model,^46^ inflammatory bowel disease,^47^ etc., make hydrogel-based 3D models hold the highest potential as a pre-clinical testing platform for drug discovery and biological performance screening.

Previously, numerous hydrogel-based cancer models have been fabricated and applied for drug screening, which showed high potential for the development of precise medicine.^44,330–333^ It has been demonstrated that cell presentation way (e.g., individual cells or spheroids) within hydrogels also matters for drug treatment efficiency. To study the influences of different cell arrangements on drug efficacy, Mano et al. developed compact 3D MG-63 spherical microtumors using the liquid mulching technology, and individual cell-dispersing gelatin methacryloyl (GelMA) hydrogel platforms were also harnessed to allow these carried cancer cells or 3D microstructures to mature and screen lorlatinib drug with the optimal performance. The data demonstrated that the spheroids displayed potent invasiveness and resistance compared to the loaded-individual cell dispersion, indicating the importance of cell aggregation in drug screening for anticancer therapies.^330^ Moreover, hydrogel also can be applied for rapidly obtaining micro-3D multi-cell polymerization units. Yang et al. proposed an assembly technique for rapidly producing 3D cell cluster arrays based on the sound mechanical force, and the arrays were wrapped in micro-hydrogels to provide 3D support and sustain ECM environment. Specifically, the acoustic surface waves generated by two pairs of bifurcated finger transducers were used to build a 3D clustering array within the chip, and the array system was finally applied to the high-throughput gradient detection of drug toxicity. Results showed that the cell aggregation activity was negatively correlated with the drug concentration in the hydrogel column, and the susceptibility to drug toxicity was lower than that of the cultured cells on 2D dishes. This 3D multicellular array provided a large number of in vitro tumor models that could be directly used for downstream drug screening.^334^

Moreover, apart from cancer-related hydrogel models, varied 3D hydrogel disease models have been developed for disease progression tracing and further drug evaluation and screening. As a paradigm, in Alzheimer’s disease (AD) mouse and human brains, the kynurenic acid/IL-4 interaction was discovered in this fabricated hydrogel model, indicative of its potential ability to further study therapeutic target of AD.^335^ In another study, Matera et al. reported a dextran vinyl sulfone hydrogel simulating pulmonary interstitial, and the soft degradable matrix could support fibrogenesis compared to that of stiff or non-degradable one, which could be used for fibrosis mechanism study and antifibrotic drug discovery.^20^ Collectively, the in vitro hydrogel model for drug discovery could facilitate the development of personalized and precise medicine leveraging the eases of hydrogel engineering and cell biology interference.

### Hydrogel scaffolds for in vivo tissue engineering and disease therapy

#### Cell-free scaffold for inducing tissue regeneration and disease therapy

Bioactive hydrogel scaffolds are widely studied as a promising candidate for guiding tissue regeneration.^336–338^ One of the mostly applied fields is wound dressing because hydrogel could remove wound exudate, provide a moist environment, and stimulate and guide tissue regeneration.^339^ Moreover, in order to accelerate wound healing or anti-bacterial properties of as-prepared hydrogels, varied growth factors (e.g., basic fibroblast growth factor (bFGF) and vascular endothelial growth factor (VEGF)) or drugs could be loaded within the hydrogel.^340,341^ For instance, a composite collagen-based hydrogel scaffold containing bFGF-loaded PLGA microspheres was developed and succeeded in expediting the wound healing process.^342^ For an infected and dry wound, physicians loaded antimicrobial silver or drugs (e.g., commercial Silvasorb) within hydrogel for wound healing application.^343^

Inspiringly, hydrogels could be used to promote the vascularization process. As stated, a biomimetic PEG-based hydrogel could not only induce intricate network formation featuring capillary-like structures in vitro, but it also promotes functional blood vessel birth after there were implanted into mouse cornea, suggestive of the high pro-angiogenesis potential for further tissue engineering application.^344^ Notably, L-arginine (L-arg) is also equipped with the pro-angiogenesis potential since L-arg could be metabolized by the intracellular nitric oxide synthase to produce nitric oxygen (NO) and NO could inhibit bacterial proliferation and enhance angiogenesis via upregulating VEGF-related signaling.^345^ Hence, many researchers have developed hydrogel incorporating or grafting L-arginine.^346–348^ Hydrogels also hold a high potential for peripheral nerve injury treatment, especially for those patients with a chronic disease like diabetes.^349,350^ In this field, some researchers developed a novel thermo-sensitive heparin-poloxamer (HP) hydrogel that incorporated bFGF and nerve growth factor (NGF) for inducing nerve regeneration in diabetic rats with the crushed sciatic nerve. Excitingly, the NGF-HP hydrogel could promote Schwann cell proliferation and nerve-related structural protein expression efficiently, thereby leading to the enhanced axon regeneration and remyelination. More importantly, motor functions were restored.^349^

Furthermore, the fully interconnected porous structures of hydrogel scaffolds played important roles in bone tissue engineering due to their osteoinductive property.^351,352^ Typically, researchers constructed a 3D printing polylactic acid scaffold that immobilized oligopeptides deriving from BMP-2 (SSVPT, Ser-Ser-Val-Pro-Thr), and this scaffold could enhance BMP stability and perform functional characteristics of osteoinduction and osteogenesis.^353^ Results showed that the fabricated scaffold could promote osteogenesis in vitro and accelerate bone regeneration in in vivo rat cranial bone defect model.^354^

#### Cell-loaded scaffolds for in vivo disease therapy

The delivery of desirable cells to damaged and diseased sites for targeted therapy has gained increasing interests and holds high potentials for cell-based therapies. However, many challenges, such as low cell survival and rigid large difficulty in cell transplantation, severely hinder the progress of this field.^100^ As a 3D scaffold, hydrogels can facilitate the positioning of cells at the target site after injection and provide appropriate biophysical and biochemical cues to promote cell integration and demanded functions.^355,356^ In this section, we focus on some promising biological applications.

##### Stem cell delivery for tissue repair/regeneration

Depending on the self-renewal property, pluripotency, trophic factor secretions, and low immunogenicity of stem cells, stem cell-based therapy provides a promising strategy for different diseases, such as corpus cavernosa injury,^357^ bone defect repair,^358^ cardiac repair,^359^ cancer therapy,^360^ etc. Up to now, many stem cell-based clinical trials (Phases 1/2) have been approved and reported to acquire unexpected outcomes for disease therapy.^361^ However, there are still some challenges in advancing stem cell therapy, e.g., low cell survival rate and uncontrolled differentiation orientation. In order to address these sufferings, different hydrogels have been developed to encapsulate and expand stem cells in vitro, and further carried out in vivo implantation, during which the minimally invasive, injectable, and biodegradable properties of hydrogels were sufficiently taken advantage of. Ballios et al. developed hyaluronan and methylcellulose (HAMC)-based hydrogels for delivering retinal stem-progenitor cells into the sub-retinal space, and the hydrogels displayed good biocompatibility and optimal biodegradability, allowing the loaded cells to be distributed in the targeted site precisely and evenly. This result demonstrated that the HAMC-based hydrogels as vehicle was promising for degenerated retina treatment.^362^

Also, hydrogels were regarded as a critical contributor for regulating stem cell differentiation. Researchers found that hydrogel degradability could affect NPC stemness in comparison to stiffness within the physiological range (~0.5–50 kPa). In this case, hydrogel degradation-induced cell stemness maintenance did not rely on traditional cytoskeleton tension production but on the enhanced cadherin-mediated cell–cell contact and β-catenin signaling.^17^ Additionally, stem cell-loaded hydrogels have also been explored for bone defect repair where hydrogel biomolecules would induce MSC differentiation into osteogenic phenotype. Moreover, due to the relatively weak mechanical properties of hydrogel itself, nanocomposite always been incorporated into hydrogel platform. It would provide stronger hardness and toughness, enhance osteogenesis differentiation, elevate enzyme activity and calcium deposition as well as upregulate osteogenic genes and proteins.^363^

Moreover, nowadays, the cell-free strategy also attracts numerous attention based on the concept that the secretions from bioengineered stem cells could be collected and delivered to the tissue defects, which were efficient for disease therapy.^364^ In this case, stem cells would be cultured and conditioned in vitro, followed by cells’ condition medium collection for further applications. In a 2D context, after tuning the stiffness of polyacrylamide hydrogel, stem cells presented a distinctive redox metabolism regulated by HIF1α signaling. In detail, stem cells preferred to display a rounded morphology with enhanced ROS production and activated HIF1α signaling on soft hydrogels compared to that one on rigid hydrogels. Expectedly, the conditioned medium deriving from stem cells cultured on soft hydrogels would promote angiogenesis in vitro and in vivo.^188,365^

Collectively, the combination of stem cells and hydrogels can be regarded as a promising method for tissue regeneration, but more attention should be paid to hydrogel design due to its complex influences on cell activities and fate.

##### Islet cell delivery for diabetes therapy

Diabetes are primarily caused by the absolute or relative deficiency of insulin.^366,367^ Pancreatic β-cell lines are usually responsible for regulating insulin production and secretion, especially for MIN6 cells. Hence, islet cell transplantation can be regarded as a promising treatment method for diabetes. However, the challenging problem is the low survival rate of islets due to the common β cell destruction after transplantation, which will reduce insulin storage capacity and glucose-stimulating insulin production.^368^ It has been documented that the cell-matrix interaction was critically vital for improving β cells’ survival and the characteristic glucose-stimulated insulin response.^369^ Inspired by it, Weber et al. developed a series of PEG-based hydrogels for MIN6 β-cell encapsulation and they also compared the effects of different incorporated ECM proteins (e.g., collagen type I, collagen type IV, fibrinogen, FN, laminin, and vitronectin) on cell behaviors. Results showed that protein incorporation could reduce cell apoptosis compared to the protein-free PEG hydrogels. Meanwhile, collagen type IV or laminin could assist MIN6 β-cells to secrete more insulin in response to glucose stimulation compared to the other groups. Wang et al. show that MIN6 β-cells could exhibit larger proliferation rate and higher glucose sensitivity with insulin secretion when human adipose ECM was incorporated, suggesting the importance of cell–matrix interaction.^19^ Hence, hydrogels are excellent platforms for artificial functional islet construction, which could dynamically and precisely control glucose balance in vivo and circumvent the drawbacks of long-term insulin injection.

Apart from the protein matrix effect on MIN6 β-cell behaviors, researchers also found that MIN6 β-cells were mechanically sensitive to cellular microenvironments, indicating the importance of placing cells in a physiologically related microenvironment with better results. For instance, Min6-derived β-cell clusters on a soft (0.1 kPa) 3D polyacrylamide scaffold showed elevated abilities of insulin secretion and glucose sensitivity than those on the rigid one (10 kPa). However, insulin expression could be suppressed when these cells were treated with MLC, ROCK, and β-catenin inhibitors even though they were cultured on the soft substrate, indicative of the participation of mechanosensing and β-catenin signaling in insulin production regulation.^370^

##### Hepatocytes delivery for liver regeneration

Bioengineered liver transplantation is promising for liver disease treatment, but limited to insufficient donor supply. Hepatocytes were always used for liver tissue construction because they are the main cell type (accounting for 80%) in liver tissue.^51^ However, there are still some difficulties in the field of functional liver tissue construction, e.g., maintaining the hepatic phenotype of primary hepatocytes. Accumulative data have demonstrated that 3D microstructure of cell living microenvironment is essential for the growth and functional phenotype expression of hepatocytes. Real liver tissue is a complex 3D integration of lobules with radial structures containing hepatocytes and non-parenchymal cells, and these cells or assembled tissues would interact with each other via paracrine crosstalk. Hence, a biomimetic 3D hydrogel platform deserves to be constructed to mimic real ECM and enable the multi-cell co-culture, which is specially essential for hepatocyte culturing. Fukuda et al. used GelMA hydrogel as a matrix to construct 3D leaflet-like micro-tissues. After long-term co-cultivation, the 3D leaflet-shaped microtissues that simultaneously encapsulated hepatocytes and fibroblasts maintained >90% of cell viability. Compared with the hydrogels that only encapsulated hepatocytes, the albumin secretion of co-cultured 3D microtissues improved the liver function, holding a high application potential in liver tissue engineering and regenerative medicine.^371^

Furthermore, hydrogel property is an important concern that needs to be taken into consideration when investigating hepatic progenitor cells (iHEP) differentiation. Cha et al. engineered a hepatic tissue via encapsulating inducible iHEP derived from recombinant fibroblasts into hydrogels with tunable mechanics and microarchitecture.^372^ Therein, iHEPs displayed enhanced viability and proliferation within hydrogels with microchannels. Intriguingly, the expression levels of albumin and CYP1A2 and hepatocyte-specific protein markers associated with mature hepatocytes were determined by hydrogel stiffness. In another independent study, the mechanical properties of hydrogel substrate were validated to determine the phenotypic characteristic maintenance of hepatocytes. Specifically, hepatocytes displayed muted hepatocyte nuclear factor 4 alpha (HNF4α) and albumin expression levels on liver ECM-mimicked HA-based substrate with a stiffness of 4600 Pa on day 7 compared to hydrogel scaffolds with lower stiffness (600 and 1200 Pa).^373^

Collectively, the hydrogel-based artificial liver could be a promising candidate for liver injury reversal. However, hydrogel properties should be taken into serious consideration when designing hydrogels for liver regeneration because hydrogel properties directly decide the behaviors and fate of loaded hepatocytes.

##### EC encapsulation for vascularization

As far as we know, vascularization deficiency will bring about serious side effects on the birth and evolution of tissues due to the deficit of adequate supplies of gas, nutrients, signaling molecules, and cells throughout the human body. Up to now, hydrogel materials have shown great potentials in guiding and inducing neovascularization in vitro and in vivo via providing physical supports and physiochemical cues. In detail, hydrogels could help ECs recall evolutionary memory and enable them to assemble into micro-capillary networks within 3D hydrogel, which was beneficial for the integration of engineered tissues after implantation.^52,374^ Campbell et al. developed an alginate-based hydrogel incorporating lyase for EC delivery. The EC-loaded hydrogels could promote new blood vessels generation on an in vivo evolving chicken egg via the interaction between ECs and chick chorioallantoic membrane (CAM) after implantation.^375^ Moon and co-workers have constructed a fantastic PEG-based hydrogel with integrin-binding sites and MMP-sensitive degradation sites, which could induce in vivo blood vessels sprouting in hydrogel after they were implanted into mouse corneas. More importantly, these blood vessels permitted Dextran-Texas red perfusion, indicating the recovery and re-modeling of functional characteristics of new vessels.^344^ Moreover, more and more smart hydrogel scaffolds have been reported for blood vessel regeneration, and found that the microenvironmental cues (e.g., material viscoelasticity, hypoxia, etc.) regulated the neovascularization process via varying signaling cascades of ECs such as cell contractility-mediated integrin β1 clustering and HIF1A signaling.^145,295^

Actually, the biomedical application of hydrogels and EC-loaded hydrogel products has a long way before their clinical translation, but some pre-clinic trials have encouraged us. For instance, researchers fabricated a skin graft using fibrin-collagen hydrogel, wherein adipose stromal vascular fraction (SVF)-derived EC population was encapsulated for expediting wound healing on five diabetic patients. The other five patients treated with nonvascularized skin grafts served as control. Results demonstrated that the hydrogels were suitable for organotypic skin cell culture and could promote wound healing with increased skin thickness and vascularization, suggestive of the potential of hydrogels for further random clinical trials and clinical translation.^376^ VentriGel, a porcine cardiac ECM hydrogel, was also tested after transendocardial injections into 15 patients. Specifically, the hydrogel was demonstrated of good biocompatibility, and could recruit vascular cells and stem cells and allow their infiltration and proliferation in vitro, which was advantageous for cell-based regenerative medicine.^377^

##### Immune cell delivery for cancer immunotherapy

Cell-based immunotherapy specially refers to the strategy that injectable hydrogels serve as a container of immune cells (e.g., T cells, NK cells, DCs, and macrophages), and concurrently allow the inhabited cells to expand in vitro and then deliver them into the tumor in vivo for repressing tumor growth and metastasis. Currently, it has emerged as a promising and efficient strategy for many cancer therapies compared to the traditional direct cell injection method.^61^ For example, Yang et al. developed a peptide-based hydrogel vaccine composed of anti-PD-1 antibodies, DCs, and tumor antigens. After subcutaneous injection, the encapsulated DCs cells show high viability and excellent antigen capture and presentation functions. Importantly, the hydrogel vaccine could recruit numerous host DCs and promote DCs accumulation within lymph nodes, followed by leading to antitumor T-cell immunity stimulation and activated CD8+ effector T cell infiltration in the tumor, which eventually resulted in the delayed tumor growth and prolonged mice survival.^55^

Macrophages can be divided into two phenotypes, namely M1 and M2 types, which show anti- and pro-tumorigenic potentials, respectively. In tumor, M2-type macrophages are dominant, and especially at the advanced-stage, they contribute to the enhanced immunosuppression microenvironment. Hence, number efforts have been made to expand M1-type macrophages and reset M2 macrophages reprogramming or polarization at tumor sites for achieving the anti-tumor effect via leveraging on material engineering.^56^ Previously, researchers constructed PEGDA and thiolated gelatin poly(ethylene glycol) (Gel-PEG-Cys) cross-linked hydrogels to encapsulate M1 macrophages for cancer treatment. Results showed that M1 macrophages-contained hydrogels could significantly activate caspase-3-induced apoptosis in HCC cancer cells, which was also contributed by upregulated nitrite and tumor necrosis factor-alpha (TNF-α) in loaded macrophages.^378^ Besides encapsulating immune cells, immune cell-regulated drugs were also entrapped and delivered by hydrogels for activating immune responses against tumor. Dai and co-workers synthesized a melittin-(RADA)6 peptide-based hydrogel to load a specific Ca^2+^/calmodulin-dependent protein kinase II inhibitor that could re-program M2 macrophages at the tumor site. After subcutaneous injection, a large number of M2 macrophages were significantly decreased, and the expression of programmed cell death protein ligand-1 (PD-L1) was increased, resulting in a vulnerable tumor microenvironment (TME) against subsequent chemotherapy.^379^

The ex vivo expansion of T cells always suffers from low efficiency, and even worse, the obtained expanded T cells display poor functions. Therefore, it is necessary and urgent to develop appropriate strategies for constructing the activated T cell-based hydrogel cancer vaccine. Previously, researchers developed lipid bilayers coated on paracrine cue-loaded mesoporous silica micro-rods for mimicking natural antigen-presenting cells (APCs). Interestingly, compared to the commercial expansion beads (Dynabeads), the fabricated scaffolds could induce twofold to tenfold polyclonal and antigen-specific expansions of T cells.^53^ In the future, other similar hydrogel-based APC-mimetic scaffolds could be also explored as novel cancer vaccines. Moreover, CAR-T cell delivery using hydrogels for tumor immunotherapy has also attracted increasing attentions.^380,381^

### Smart hydrogels for drug delivery and targeted therapy

Hydrogels have prominent advantages for drug delivery, especially for those hydrophobic drugs and macromolecule cargos. For instance, the bioavailability of hydrophobic drug like zinc phthalocyanine (ZnPC) is always quite low due to their poor solubility in physiological condition, and they are disabled to be used alone. Given that, suitable delivery system is required to enhance their local drug concentration and augment their therapeutic efficiency. As reported, Ji and co-workers have fabricated a thermal sensitive PCL-PTSUO-PEG hydrogel for ZnPC encapsulation,^382^ and a high loading efficiency (>85%) was reached. More excitingly, the local concentration of water insoluble ZnPC (<0.1 mg/mL in water) exceeded 1.9 mg/mL, which outperformed other drug delivery systems such as liposome in significantly improving its photobiological activity.^382^

In particular, various smart hydrogels that could respond to various external or internal stimuli have been developed and extensively investigated, which show high potentials in drug delivery for disease therapy and concurrently overcome the shortcomings of traditional drug carriers.^383,384^ Moreover, some smart hydrogels are beneficial for living circulating cancer cells identification and isolation in a noninvasive manner.^385,386^ Generally, hydrogels can be engineered and endowed with various physiologically responsive properties and complete sol-gel transition when injected into the tumor site, which could improve the accuracy of dose manipulation and therapeutic drug distribution, and augmented drug permeability in tumor tissues. Very recently, hydrogels have been explored and employed in immune checkpoint blockade therapy against various tumors,^387^ even for cold tumor.^388^ Additionally, some smart nucleic acid-based hydrogels also show great potential for non-invasive tumor PTT. Zhang et al. designed a PDA-coated nucleic acid nano-gel as a siRNA-mediated therapeutic complex for low-temperature PTT (42–45 °C).^389^ Differing from cell necrosis induced by high temperature in traditional PTT (>50 °C), the hypothermia PTT relied on a safe cell apoptosis pathway to eliminate tumor cells. Considering that the heat-shock-protein 70 (HSP70) can alleviate the cell damages caused by hyperthermia,^390^ this study firstly used HSP70-targeted siRNA to graft with polyhexyl ester HNA-g-PCL and introduced them into nano-hydrogel particles. Then, the obtained nano-gels entrapping siRNA were coated with a layer of PDA to protect them from enzymatic degradation and mediate the photothermal transformation under near-infrared (IR) light. After surface PEG, the complex demonstrates the robust capability of effectively ablating tumors under relatively mild conditions.

Hydrogels with multi-functions have attracted more attention due to its synergistic therapeutic consequences.^391,392^ Liu et al. proposed a new light-induced in situ hybridization hydrogel system to realize the powerful photodynamic and immune combined therapy against tumors.^9^ This system consisted of catalase modified with photosensitizer and PEGDA as a polymer matrix. Furthermore, immune adjuvant nanoparticles were introduced into this system to further reinforce antitumor immune responses after photodynamic therapy (PDT). This study found that ROS production under red light irradiation via PDT pathway could trigger the polymerization reaction and cause rapid in situ gelation of the Ce6-CAT/rpnps/PEGDA hybrid solution at the injection site. Results showed that the composite hydrogel system could not only achieve repeated treatment of PDT but also could continuously regulate TME (such as continuous hypoxia mitigation), which would be beneficial for PDT and immunotherapy. In several cases, multiple rounds of gel-based PDT could generate multiple waves of tumor-associated antigen exposures due to the prolonged retention of immune adjuvant RPNP in tumor, thereby inducing stronger antitumor immune responses. After this treatment, researchers observed that the mice gave birth to considerably elevated immune memory effects in the mice, which effectively protected the mice from re-attack by cancer cells.

### Other applications

Apart from the above-mentioned application practices, hydrogels could be also extended to other biomedical fields, such as tissue fillers,^393^ bioimaging,^394^ biosensor,^395^ conductive wearable/implantable biodevices,^66,67^ soft robots,^396^ etc. Hydrogel products used for cosmetic filler have been widely reported and evaluated, where HA gel is the mostly used. Additionally, hydrogels have also been explored as intraocular vitreous body filler to provide physical support and barrier function.^397^

Meanwhile, hydrogel-based bioimaging furnished an important tool to assist the diagnosis, assessment and therapy of various diseases because it is favorable for real-time monitoring of cell behavior, tissue location, and morphology. Generally, various imaging modalities could be introduced into hydrogel-based imaging systems, e.g., computed tomography (CT), single-photon emission computed tomography, ultrasound,^398^ magnetic resonance imaging (MRI),^399^ fluorescence imaging,^400^ and positron emission tomography. However, hydrogels share approximately identical atomic composition with living tissue except bone, which will result in undifferentiated x-ray absorbances. Hence, different imaging agents are usually incorporated or conjugated within the hydrogel network, which will specifically respond to disease biomarkers theoretically. For instance, Tan et al. developed a thermosensitive simvastatin/Poloxamer 407 hydrogel, which could be delivered to the desired disease via CT-guided percutaneous intraosseous injection. This special hydrogel could result in increased bone mineral density and microstructure, and show highly promising for osteoporosis treatment and fragile fracture prevention. This phenomenon was believed to result from the upregulated VEGF and BMP-2 within the disease microenvironment.^401^ Tondera et al. reported a self-healable and injectable hydrogel based on the peptide-oligosaccharide noncovalent interactions. After injection into the immunocompetent mice, the release profile of encapsulated compounds in hydrogel has been recorded continuously for 9 months via MRI and optical imaging using an IR fluorophore, and no adverse inflammatory responses were observed and good stability was obtained. Fluorescence imaging has been exemplified by many experiences to hold great promising for monitoring drug delivery and hydrogel behavior in vivo after introducing sensitive fluorescent conjugates.^402^ Zhang et al. designed a type of Dextran-methacrylate/carboxymethyl nanocrystalline cellulose hydrogel that incorporated nanoparticles, and this hydrogel could real-time monitor and predict hydrogel degradation.^403^ Specifically, aggregation-induced emission (AIE) molecules (tetraphenylethene) were grafted onto the surface of mesoporous dopamine and then were incorporated into hydrogels, endowing the as-synthesized hydrogels with fluorescence imaging.^403^ Additionally, the treatment modality of AIE via elevating ROS production was also added to obtain the all-in-one theranostic system.^111^

Hydrogels that are used as biosensors have also elicited a surge of interests.^404,405^ Generally, a biosensor consists of four main compartments, including bioreceptor, base material, transducer, and electric system. Usually, hydrogels acted as the base material on which the bioreceptors (e.g., enzyme, antibody, cell, etc.) were deposited or entrapped to interact with the analyte, followed by a conversion from biochemical reaction-produced signals to measurable signals.^406^ As a paradigm, Crulhas et al. synthesized a PEG diacrylate hydrogel that incorporated ferrocene-coupled superoxide dismutase for monitoring reactive oxygen species within the TME. Sometimes, hydrogels can be also utilized for biological event detection at the absence of bioreceptor because hydrogels were featured of the tunable swelling property after biological interaction-induced stimulation, such as pH, mechanical pressure, etc.^407^

As the multidisciplinary integration and fusion move forward to a comprehensive and deep state, hydrogels enter a newly emerging territory, i.e., conductive wearable/implantable devices.^66,67^ Currently, there are two strategies for conductive hydrogels construction including electronic conduction via conductive filler (e.g., metal, Mxene, conductive polymers, etc.) incorporation and free motion ions incorporation (e.g., Li^+^, Na^+^, Fe^3+^, etc.). Moreover, in order to elevate the performance of conduction hydrogels, different conductive hydrogels with distinct properties, such as excellent bio-adhesive property, high electrical conductivity, toughness, stretchability, etc., have be developed with encouraging results. For instance, some researchers reported an orthogonal photochemistry-assisted printing strategy to fabricate tough conductive hydrogels (toughness up to 10^3^ MPa), which were mainly composed of EDOT monomer and tyramine-modified poly(vinyl alcohol). Results demonstrated that the photopolymerization during the printing process was fast (*t*_gel_, ~30 s) and highly controllable for arbitrary structures construction.^408^ Moreover, in another study, a polyacrylamide–alginate hydrogel was developed, which exhibited an enhanced electrical conductivity (>350 S cm^−1^) compared to traditional hydrogel products (<100 S cm^−1^). Concurrently, the developed conductive composite could maintain soft compliance (Young’s modulus <10 kPa) and deformability even though silver flakes were incorporated.^409^ More interestingly, an ultra-rapid responsive manipulator consisting of a thermo-responsive hydrogel layer and an electric heater layer has been constructed based on the cephalopod’s suction cup working mechanism, which enabled the manipulation and transportation of live cells/tissue sheets.^410^

## Current advances and challenges of hydrogels in clinical or pre-clinical applications

**Fig. 11:**
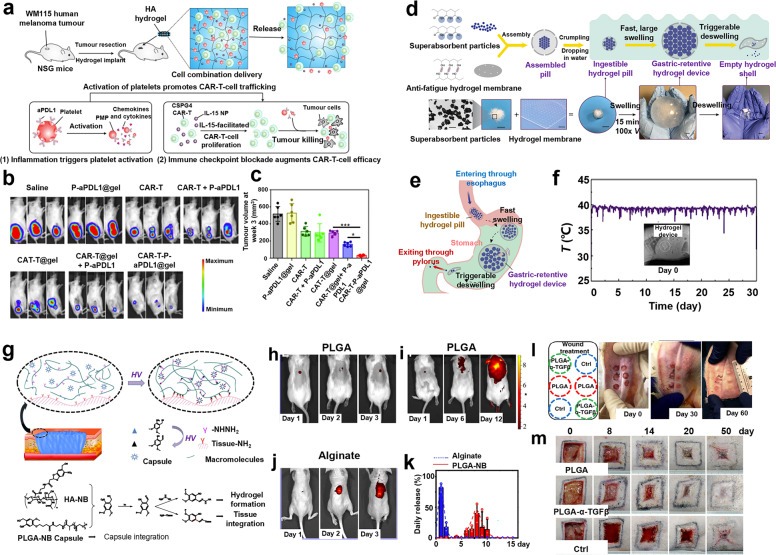
The preclinical studies of hydrogel applications. **a**–**c** Preparation of a hyaluronic acid (HA)-based hydrogel that released CAR-T cells to target the human chondroitin sulfate proteoglycan 4 (**a**) and anti-PDL1-conjugated platelets for inhibiting post-surgery melanoma tumor recurrence (**b**, **c**). *p = 0.0486, ***p < 0.001^380^ Copyright 2021, Springer nature. **d**–**f** Pufferfish-inspired ingestible hydrogel device (**d**, **e**) for long-term gastric retention and physiological monitoring like porcine gastric temperature (**f**).^411^ Copyright 2019, Springer nature. **g**–**m** A photoinduced imine-cross-linking hydrogel (**g**) with pulsatile TGF-beta inhibitor release characteristic (**h**–**k**) for promoting scarless wound healing in rabbit ear scar (**l**) and porcine skin (**m**).^339^ Copyright 2021, Springer nature

Due to the tunable physiochemical properties and favorable biocompatibility, hydrogels have been widely investigated and applied for clinical purposes. The most well-known FDA-approved application fields of hydrogels are facial correction/esthetic, filler, contact lens, etc. Nowadays, hydrogel products show emerging potential for various tissue regeneration and disease treatment. According to the Clinical Trials.gov database (https://clinicaltrials.gov/), there are 184 completed and some recruiting clinical trials for application tests of different hydrogel product worldwide in sum. Generally speaking, the current application domain of hydrogels focuses on traditional skin diseases, eye diseases, diabetes, brain and neuro diseases. Moreover, numerous inspiring data from preclinical studies have demonstrated the broad application window of hydrogels (Fig. 11). For instance, Gu and co-workers have fabricated a HA-based hydrogel reservoir used for encapsulating CAR-T cells and anti-PD-L1-conjugated platelets, which could efficiently inhibit post-surgery melanoma tumor recurrence (Fig. 11a–c).^380^ The encapsulated CAR-T cells displayed good survivability, proliferation, and bioactivity for targeting human chondroitin sulfate proteoglycan 4 that was selectively highly expressed in melanoma cells rather than healthy cells. After tumor excision, the inflammation at tumor site induced the activation of loaded platelet from hydrogel, and resulted in PD-L1 antibodies release from platelets via forming platelet-derived microparticles, and simultaneously resolved the immunosuppressive TME. In another independent study, researchers developed a pufferfish-inspired ingestible hydrogel device with good biocompatibility, which could be a desired non-invasive alternative to implantation (Fig. 11d–f).^411^ Due to the high swelling ratio, swift swelling speed and long-term robustness, the hydrogels could achieve long-time gastric residency. Unexpectedly, the hydrogel device could retain in porcine stomach for more than one month and thus could continuously and steadily monitor the temperature via the carried sensor. Another exciting research work focused on scarless wound healing using photoinduced imine-cross-linked sutureless hydrogels that allowed pulsatile release of encapsulated TGF-beta inhibitors.^339^ Therein, o-nitrobenzene (NB) was conjugated on PLGA polymer, and PLGA capsules with thick shell and hollow structure for loading and pulsatile release of TGF-beta inhibitor were within easy reach. Meanwhile, partially NB-modified HA was served as pre-gelling polymers to deliver PLGA-NB capsules. The animal data from rabbit ear wound and porcine skin wound demonstrated the efficiency of fabricated hydrogel dressing for scarless wound healing (Fig. 11g–m).

More inspiringly, more and more novel hydrogel developments are ongoing so as to cater to the ever-increasing demands of the market and patients. Typically, novel DNA hydrogels have been developed recently via a microfluidic platform. They could achieve rapid diagnosis (15 min) of severe acute respiratory syndrome coronavirus 2, consequently reducing the economic burden on a global scale.^412^ Collectively, hydrogel product development is beneficial for human beings’ health and social and economic development, and more efforts need to be made toward the lab-to-clinic transition and advanced applications of hydrogel products.

Up to now, there are still some challenges and unresolved technical problems that limited the further applications of hydrogels. We herein discussed the unresolved challenges and potential development directions in the following four aspects to facilitate hydrogels-based researches to move forward toward biomedical application in the future.

1. Potential side effects and long-time efficiency after hydrogel injection/implantation (e.g., inflammation, fibrosis, calcification, joint pain, and joint effusion). Sarojini et al. prepared a PHEMA-based hydrogel to be implanted in the rabbit cornea. Unfortunately, calcification at the site of hydrogel injection was observed after around 12 weeks post-implantation, suggestive of insufficient biocompatibility of this hydrogel for long-time application.^413^ Additionally, a majority of materials for hydrogel construction would elicit immune-mediated foreign body responses (FBR), result in fibrosis surrounding hydrogel and inhibit other cells infiltration into the hydrogel, eventually leading to the failure of treatment. Anderson et al. created a 77-member library of modified alginate analogs, in which three triazole-contained alginate analogs showed neglectable fibrotic responses in primates, indicating that these types of synthesized materials could efficiently evade FBR for further in vivo applications.^21^ Therefore, a deep and systematic understanding of materials should be performed to facilitate their biomedical applications.
2. Injection or implantation route and minimum effective dosage. The wrong administration manner of HA hydrogels for esthetics would cause swelling, nodules, pain, and other complications for the patients.^414^ Stem cell-dependent therapies also need to be taken care. Some researchers have cultured and collected targeted allogeneic secretome and loaded them into hydrogel to shape into a cell-free therapy. Moreover, cell-encapsulated hydrogels have also been constructed and implanted at the disease site, which can be expected as an active secretome factory to continuously secrete and release factors to treat disease. However, when secretome served as a therapeutic drug, its efficiency and side effects were also dose-dependent,^415^ which, thus, need to be taken seriously when designing hydrogels objective to some certain diseases.
3. Paracrine crosstalk between cells for cell-based hydrogel application in vivo. Various cell types co-exist simultaneously within in vivo stromal microenvironment, which cooperatively work in a highly organized manner to maintain body homeostasis. When cell-loaded hydrogel was implanted into the human body, the cells within hydrogel would intricately interact with the surrounding cells via paracrine crosstalk, which may influence the eventual therapeutic effect. For instance, cancer cells could induce stem cells to differentiate into cancer-associated cells that were preferable to promote tumor activities and invasiveness.^416^
4. Dynamic stroma microenvironment. A plethora of studies has shown that the biochemical and biomechanical properties of stroma matrix in vivo keeps dynamically and finely changing, which could spatiotemporally regulate cell biology. Even though the varied dynamic systems have been developed and applied, they were usually used for drug delivery. Additionally, cell behaviors and related signaling cascades within the dynamic platform have not been completely explored yet. Therefore, there is still a long way to develop rational dynamic hydrogel systems for enhancing our understanding and cognition of cell behaviors in vivo, which is also significant and meaningful for precise medicine.

## Perspective and summary

Up to now, hydrogels have shown great impacts in many biomedical fields, such as esthetic medicine, tissue engineering, drug screening, cancer therapy, etc. The deep and comprehensive understandings of cell behavior and signaling transductions in response to physicochemical and structural characteristics of hydrogel matrix advances the ongoing development of material approaches so as to better regulate cell biology and fate for desirable demands. Generally speaking, the cell would sense the surrounding material cues and transduce these cues into intracellular biochemical signals that could affect viability, gene expression and cell lineage commitment. Importantly, in order to better recapitulate the complex native matrix, researchers focus on more multiple characteristics and dimensions of hydrogels including three-dimensionality, hydrogel architecture, degradability and dynamic properties to deepen and widen our understanding on how the physiochemical, mechanical and structural cues of hydrogels regulate the phenotype, function and fate of cells. Despite acquiring exciting and significant progress in hydrogel design, nowadays, the clinic transition of hydrogel products is limited due to the unexpected side effects and complications as well as improper administration manner. These challenges can be addressed via material optimization after material library construction and screening. Although cell-loaded hydrogels for tissue regeneration and disease treatment in vitro have been successfully demonstrated to exhibit promising therapeutic effects, the in vivo long-time efficiency is still unknown due to the complex cellular microenvironment. Therefore, a systematic and comprehensive knowledge of the interactions between cells and matrix is compulsory to facilitate their clinical applications in vivo. Also, even though varied types of dynamic hydrogels have been developed, their application domain is mainly limited to the drug delivery. Hence, the understanding of cell biological responses and signaling transductions within smart hydrogel will be beneficial to extend their applications in the future.

Overall, in this review, the systematic correlations between physiochemical, composition, or structural characteristics of hydrogels and cell biology and its involved signaling cascades were discussed and highlighted, followed by the application outline on current advances in the area of hydrogels for biomedical applications. The completely understood associations with biological responses and signaling pathways will lay the foundation to further clinical applications, and the clinical trials and current challenges will guide the way for future hydrogel design.

## Data Availability

All data included in this study are available upon request by contact with the corresponding author.
